# Supplementary material for: Pyrolytic elimination of ethylene from ethoxyquinolines and ethoxyisoquinolines: a computational study
Source: Sci Rep. 2023 Apr 17;13:6248. doi: 10.1038/s41598-023-33272-2 (PMC10110564; doi:10.1038/s41598-023-33272-2)
Supplement: Supplementary file 1 — Supplementary Information. [file 41598_2023_33272_MOESM1_ESM.docx]

**Supporting information for**

**Pyrolytic elimination of ethylene from ethoxyquinolines and ethoxyisoquinolines: A computational study**

Mohamed A. Abdel-Rahman,^1*^ Mohamed F. Shibl,^2*^ Mohamed A. M. Mahmoud^3^

^1^Chemistry Department, Faculty of Science, Suez University, Suez, 43518, Egypt

^2^ Renewable Energy Program, Center for Sustainable Development, College of Arts and Sciences, Qatar University, 2713 Doha

^3^Basic Sciences Department, Tanta Higher Institute of Engineering and Technology, Tanta 31511, Egypt.

*Corresponding author:

E mail: *Corresponding authors: E-mail: Mohamed.Abdel-Rahman@sci.suezuni.edu.eg (Mohamed A. Abdel-Rahman), [mfshibl@qu.edu.qa](mailto:mfshibl@qu.edu.qa) (Mohamed F. Shibl)

| 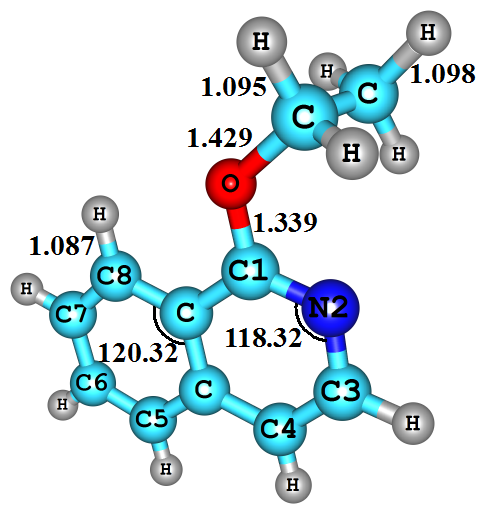**1-EisoQ** | 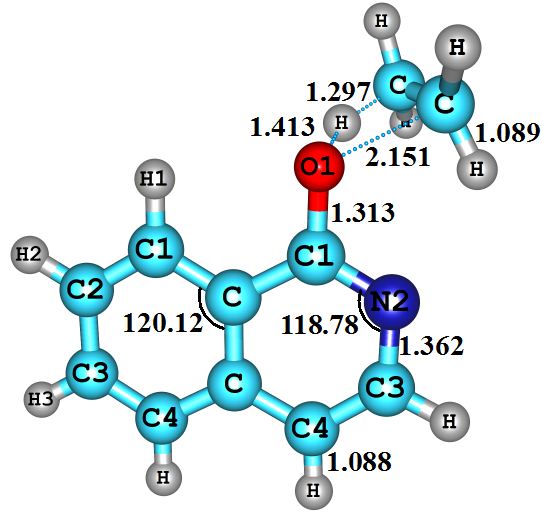**TS1_enol_** | 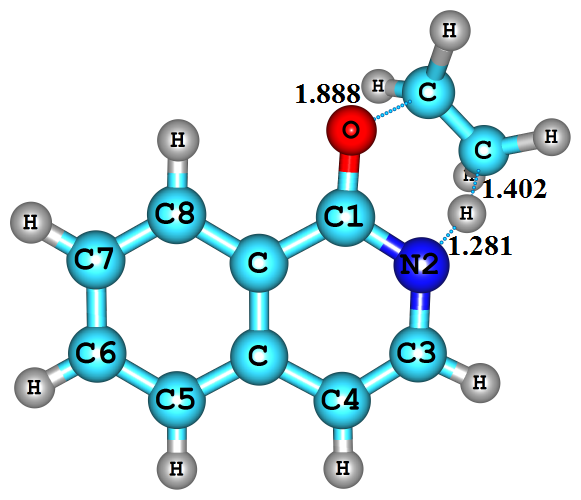**TS2_keto_** | 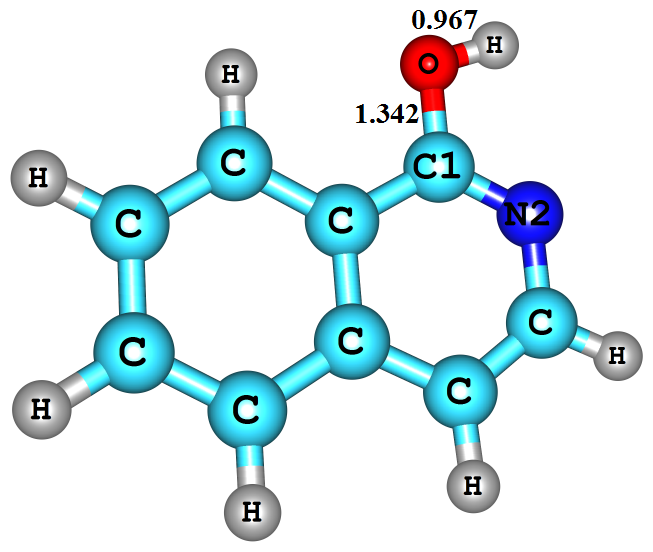**1-OHisoQ** |
| --- | --- | --- | --- |
| 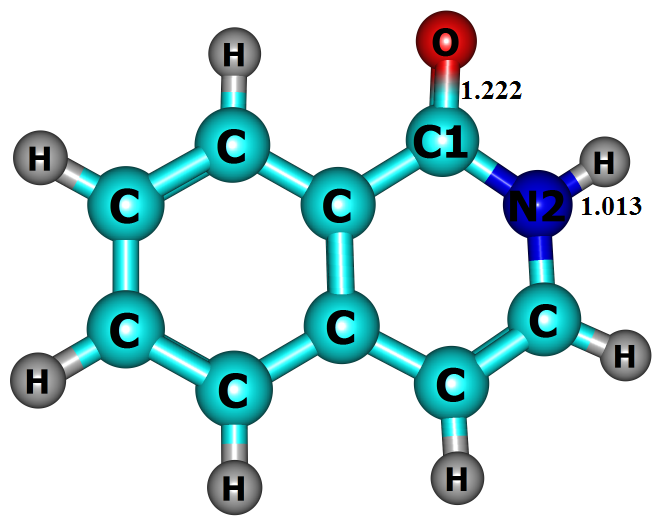**1-NHisoQ** | 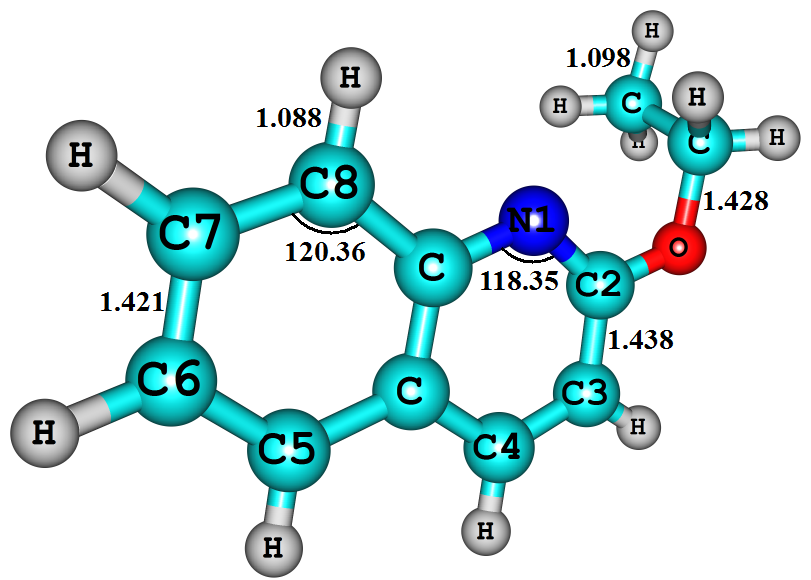**2-EQ** | 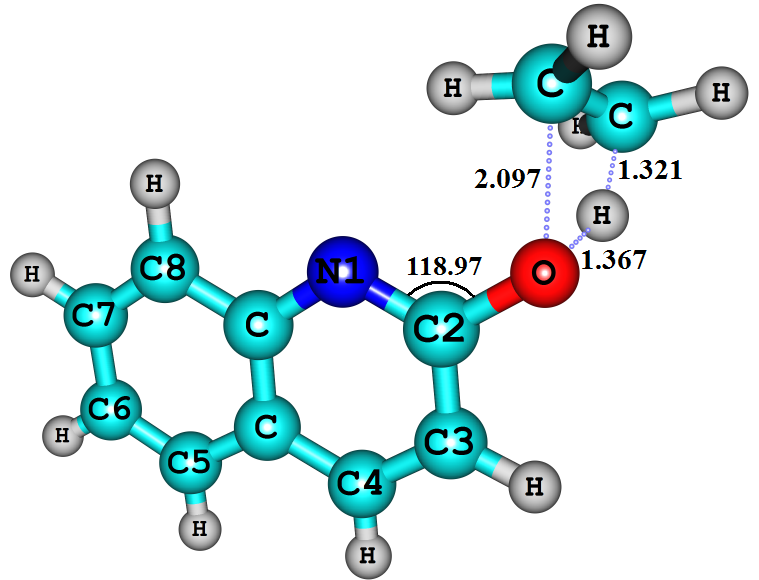**TS3 _enol_** | 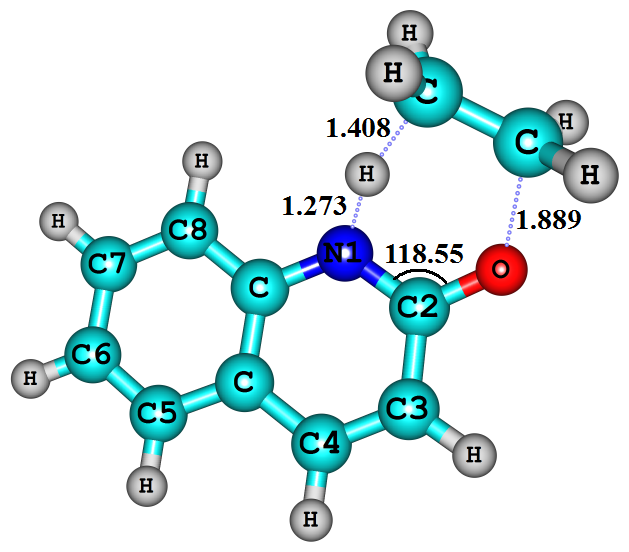**TS4 _keto_** |
| 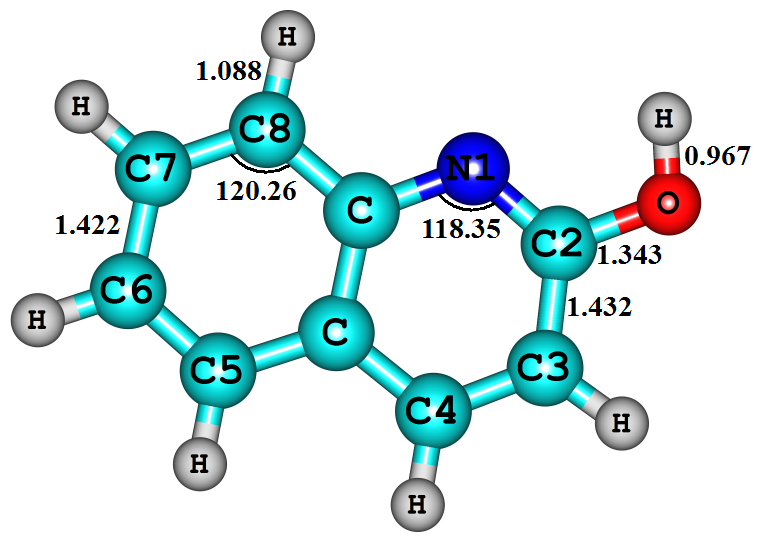**2-HOQ** | 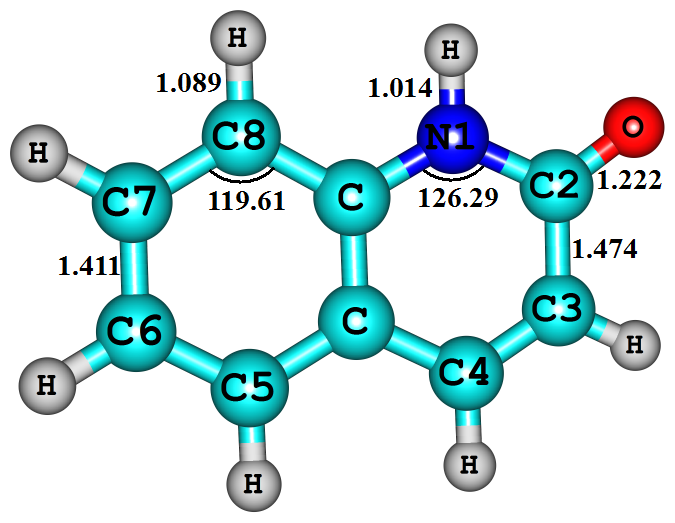**2-OQ** | 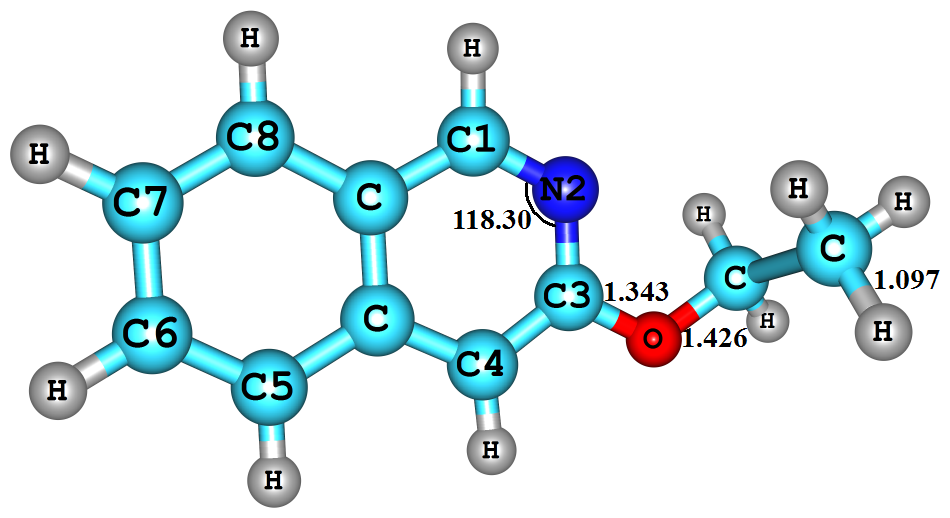**3-EisoQ** | 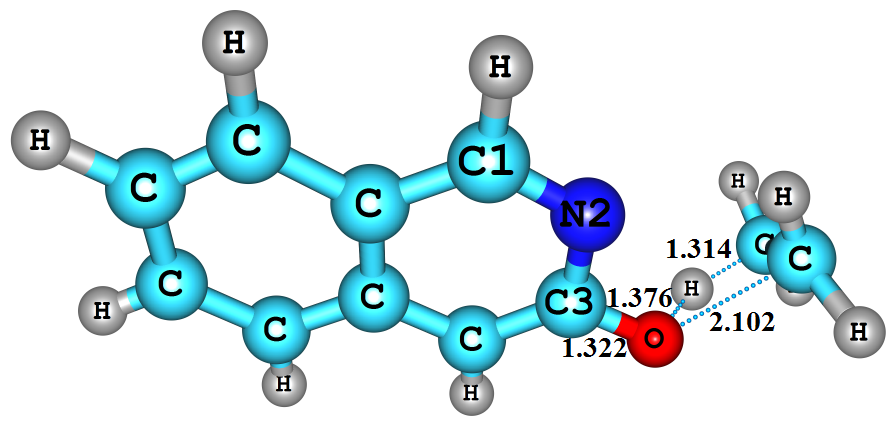**TS5 _enol_** |
| 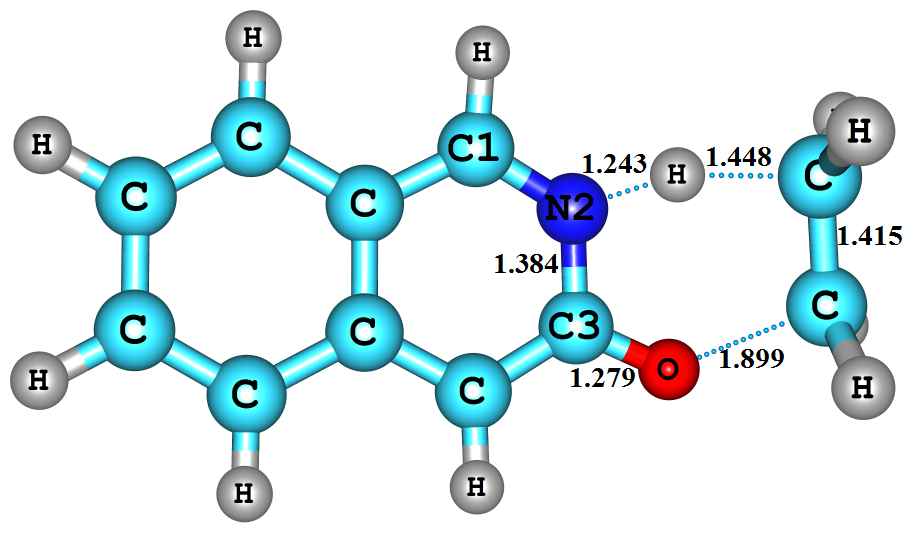**TS6 _keto_** | 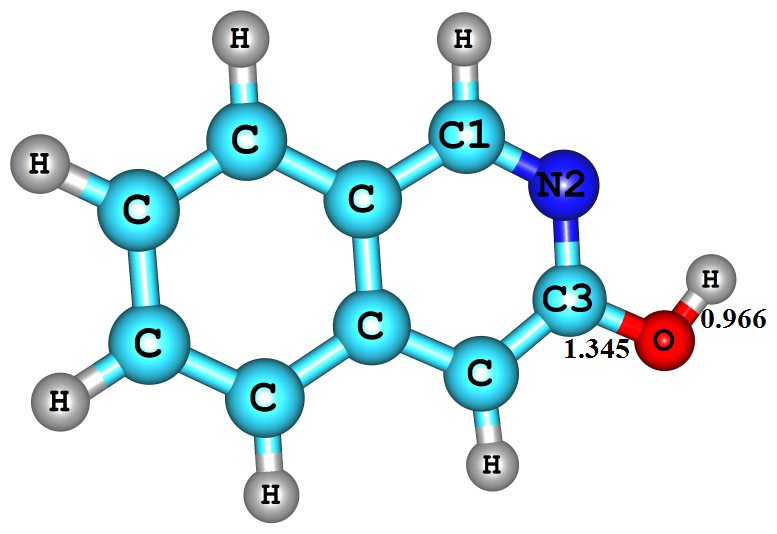**3-OHisoQ** | 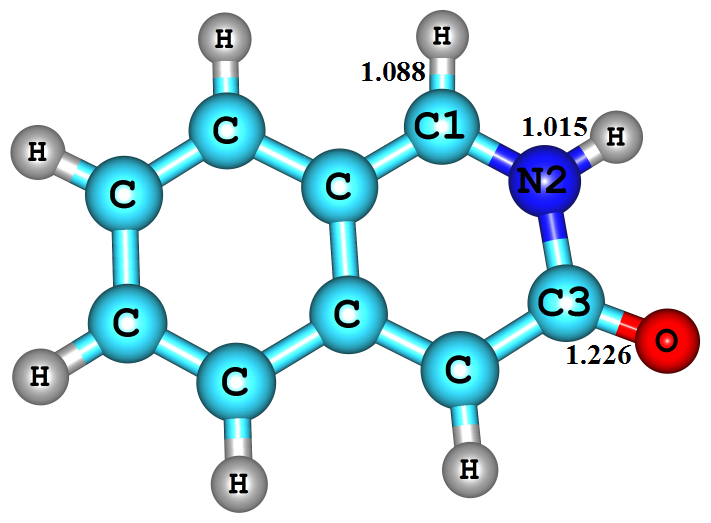**3-OisoQ** | 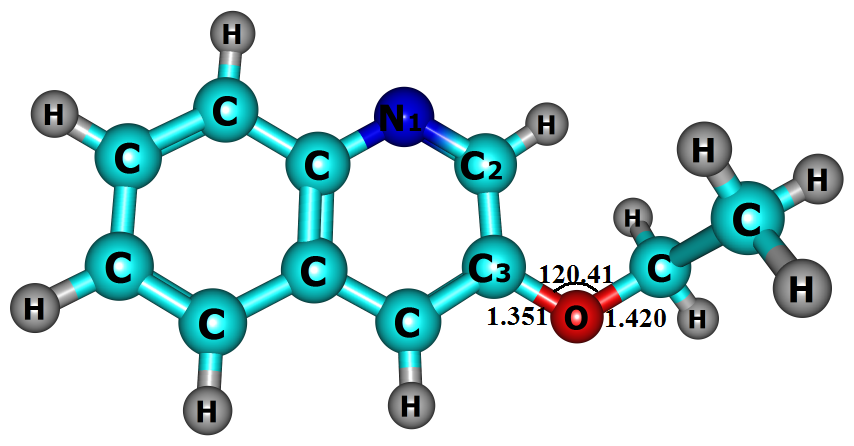**3-EQ** |
| 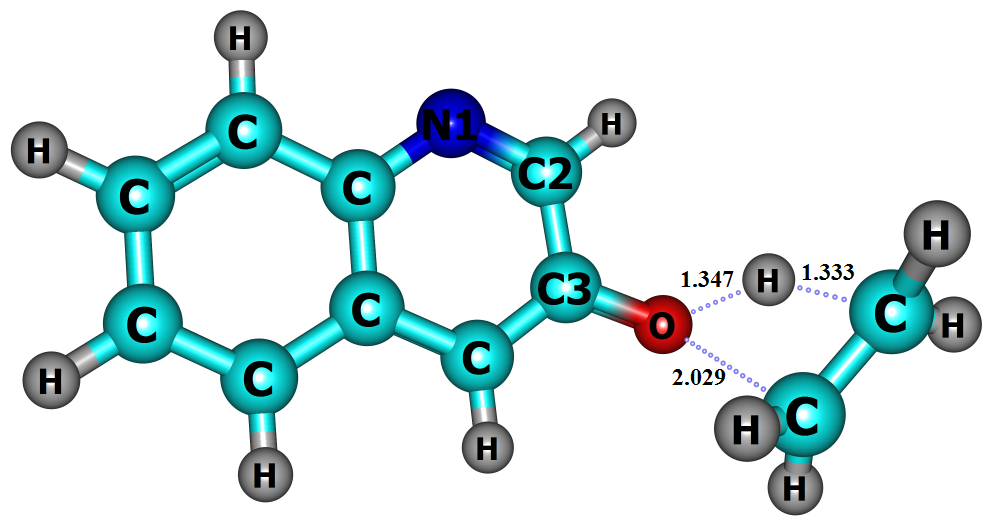**TS7_enol_** | 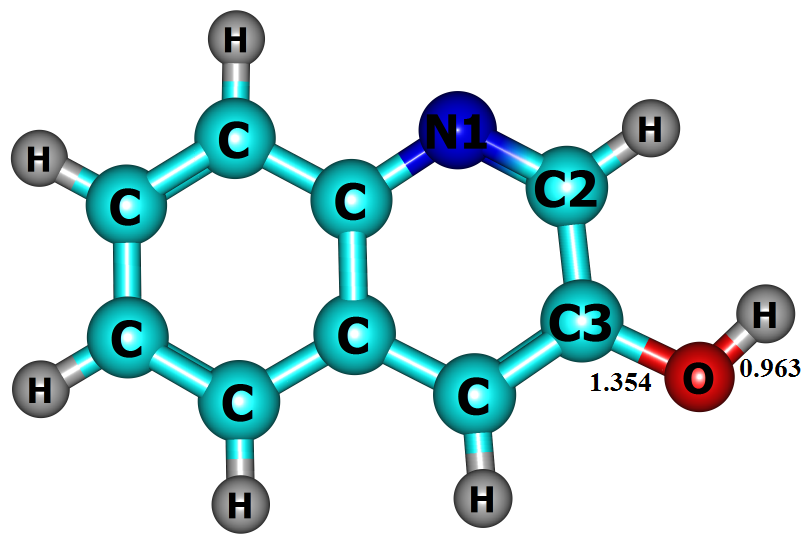**3-OHQ** | 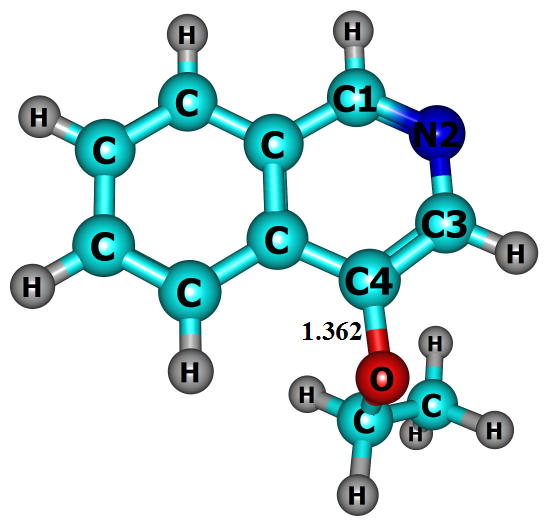**4-EisoQ** | 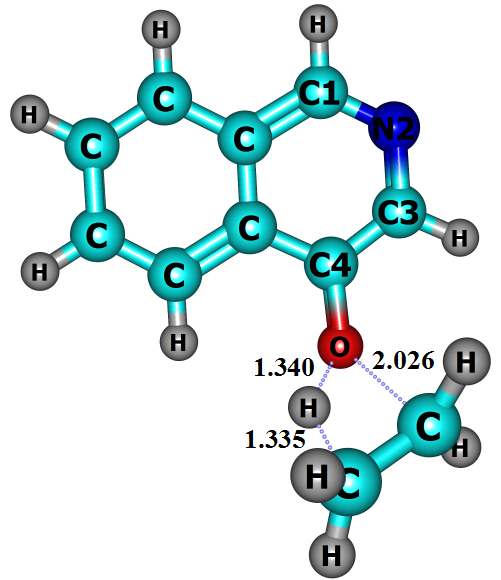**TS8_enol_** |
| 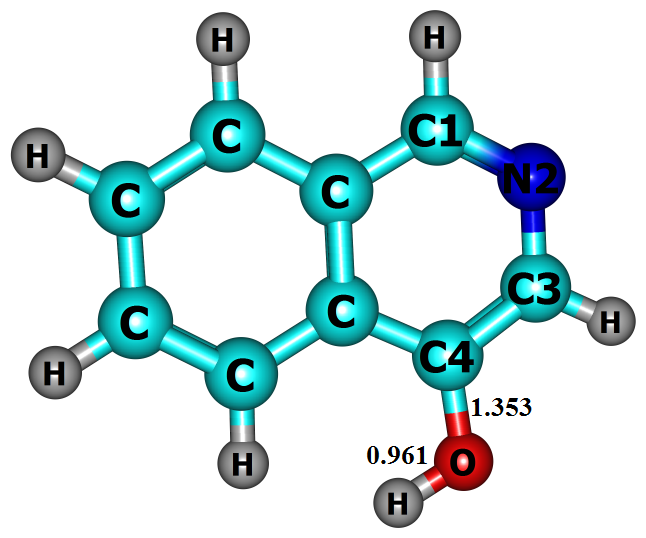**4-OHisoQ** | 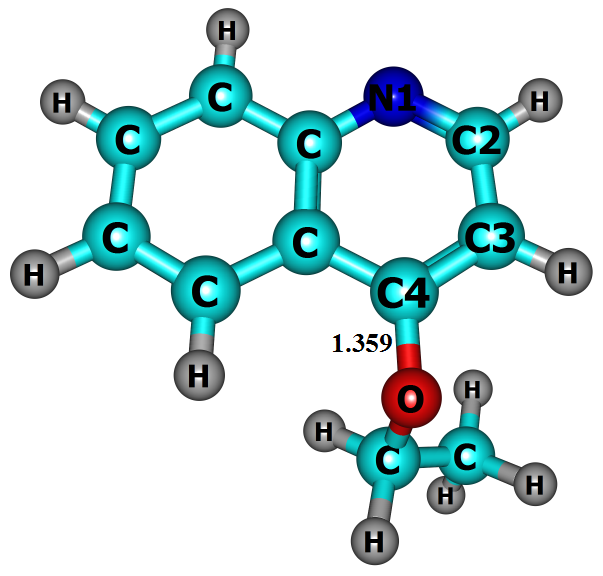**4-EQ** | 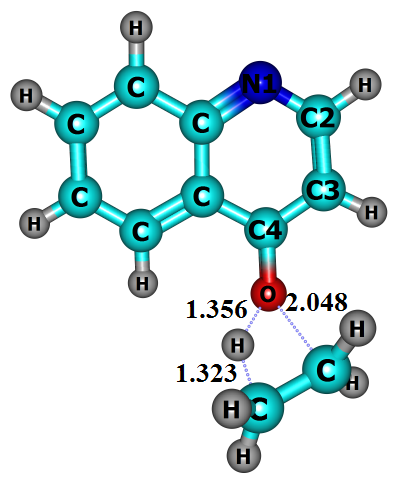**TS9_enol_** | 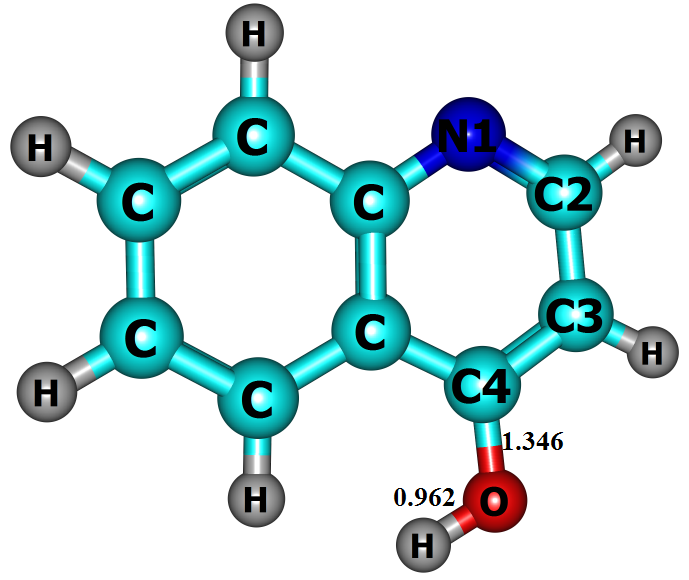**4-OHQ** |
| 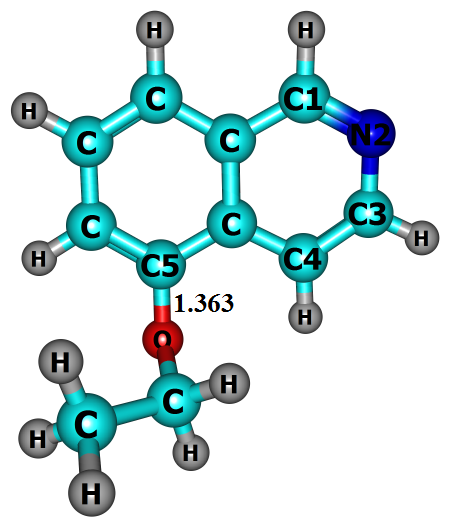**5-EisoQ** | 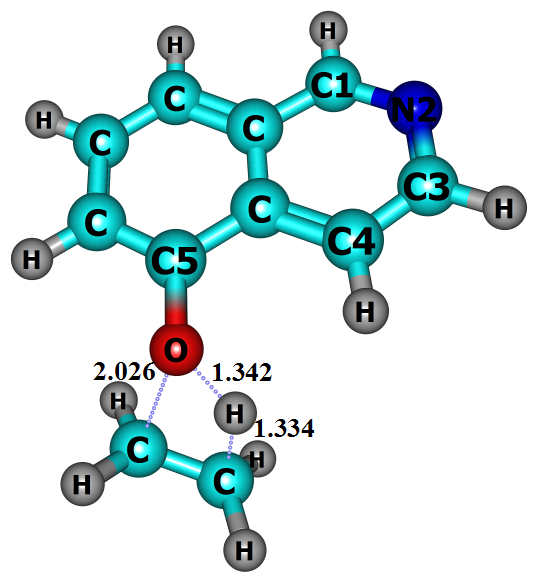**TS10_enol_** | 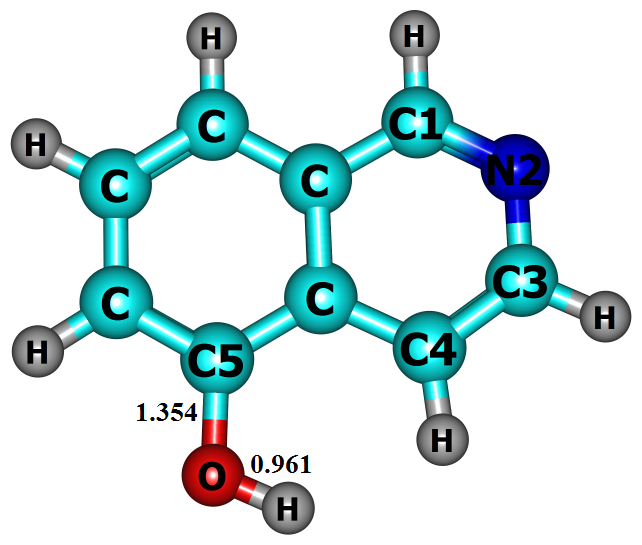**5-OHisoQ** | 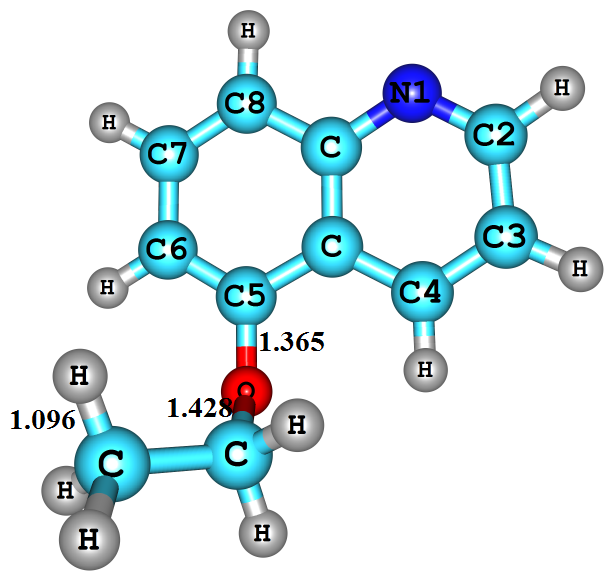**5-EQ** |
| 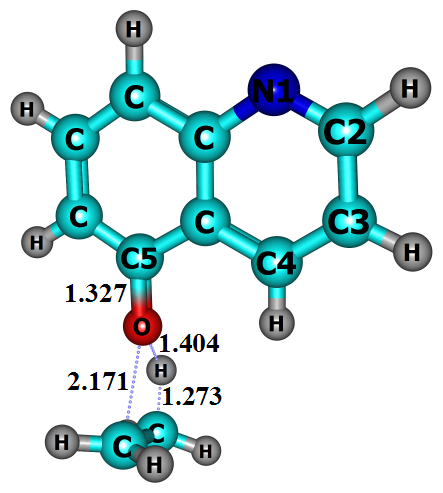**TS11_enol_** | 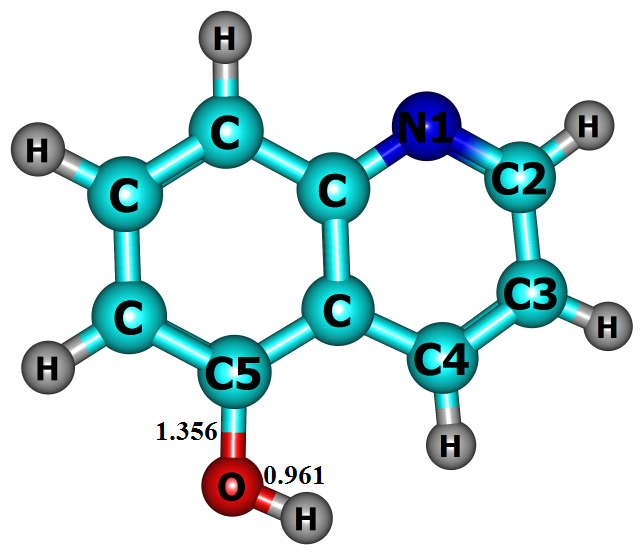**5-OHQ** | 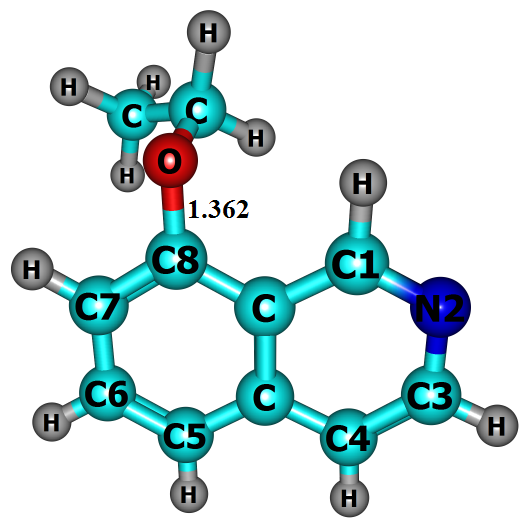**8-EisoQ** | 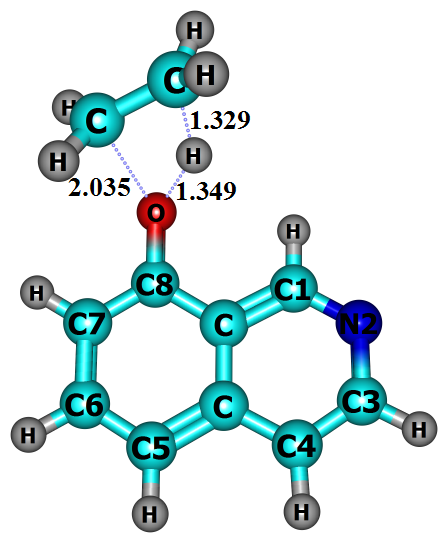**TS12_enol_** |
| 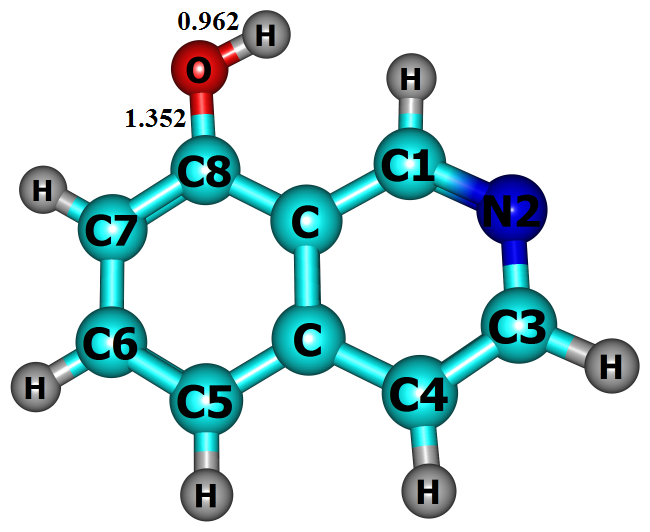**8-OHisoQ** | 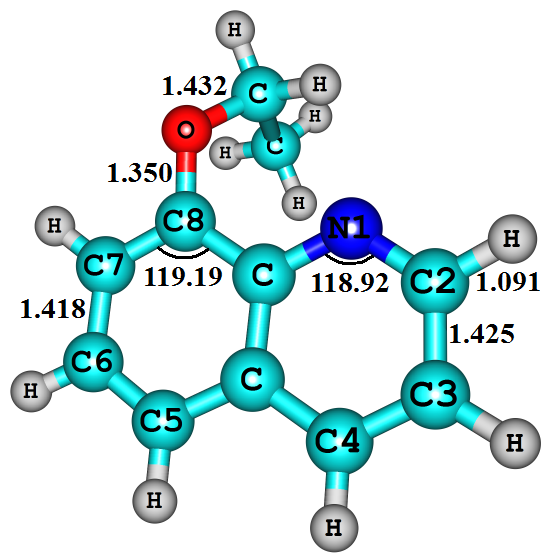  **8-EQ** | 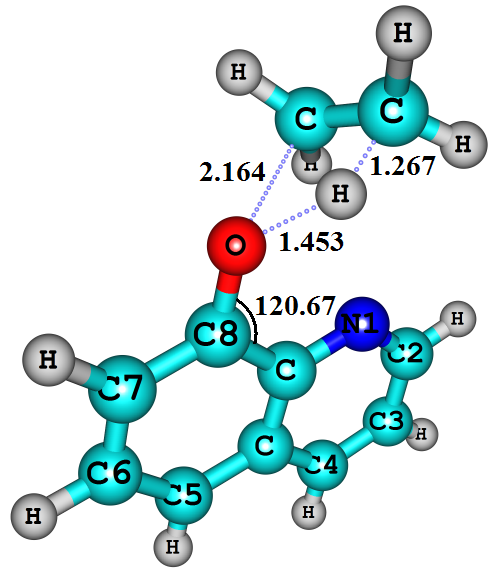**TS13_enol_** | 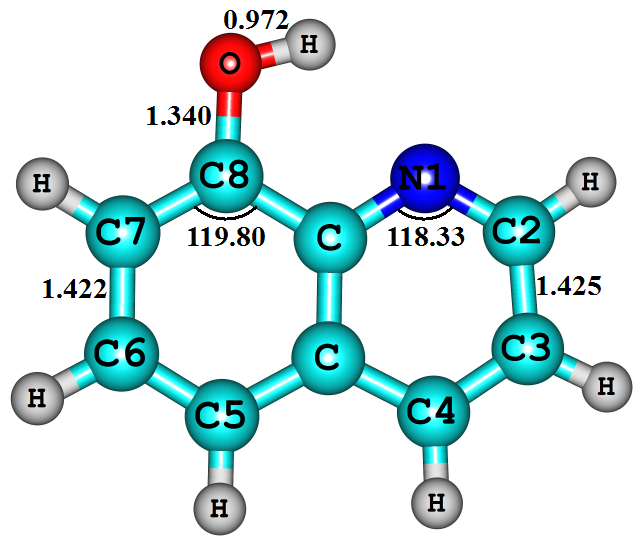**8-OHQ** |
| 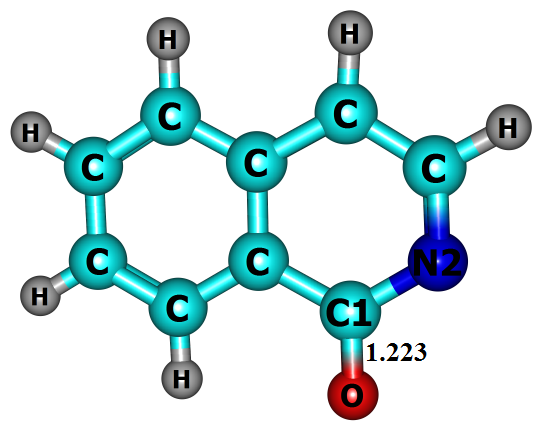**1-iso**^•^**OQ** | 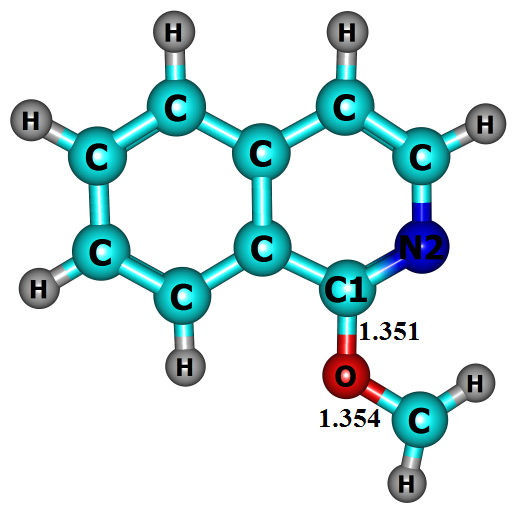**1-^•^CH_2_OisoQ** | 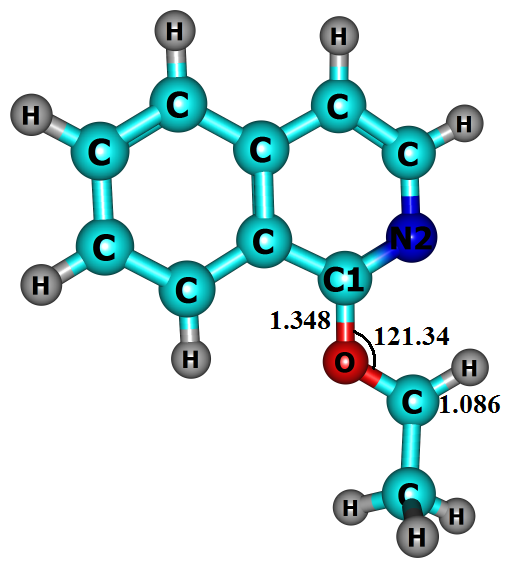**1-CH_3_^•^CHOisoQ** | 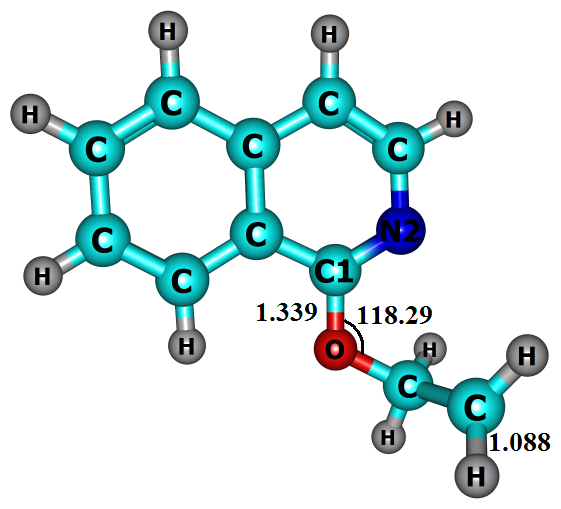**1-^•^CH_2_CH_2_OisoQ** |
| 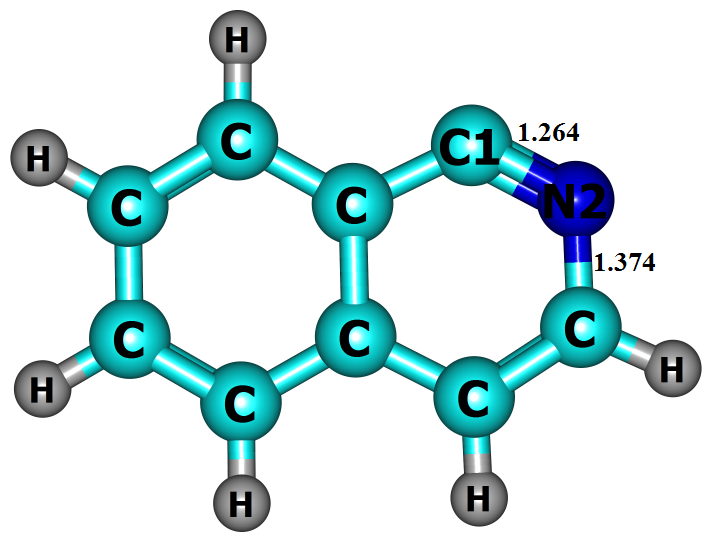**1-isoQ^•^** | 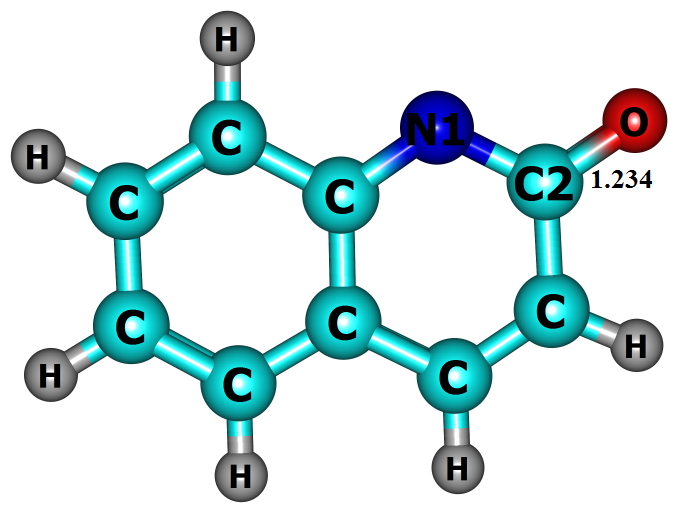**2-^•^OQ** | 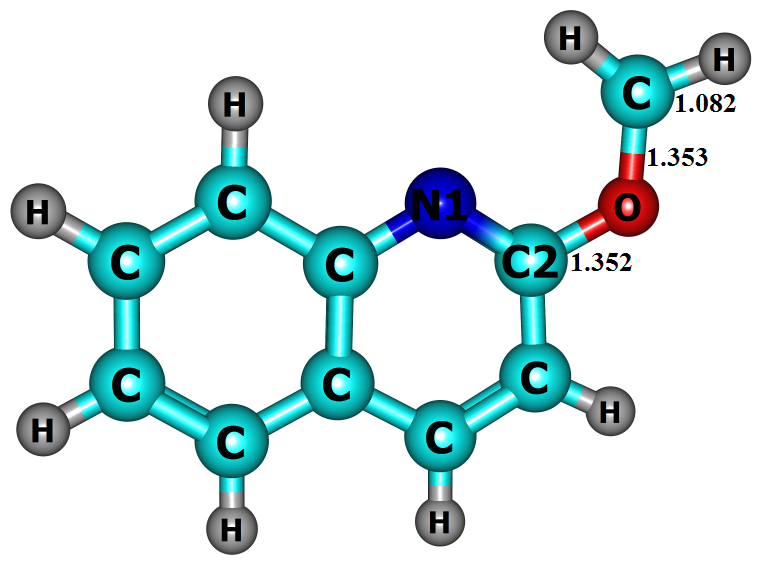**2-^•^CH_2_OQ** | 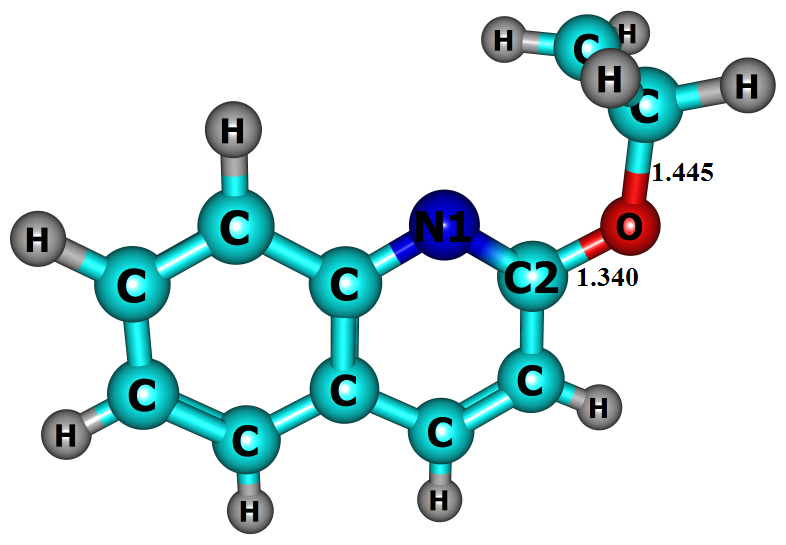**2-^•^CH_2_CH_2_OQ** |
| 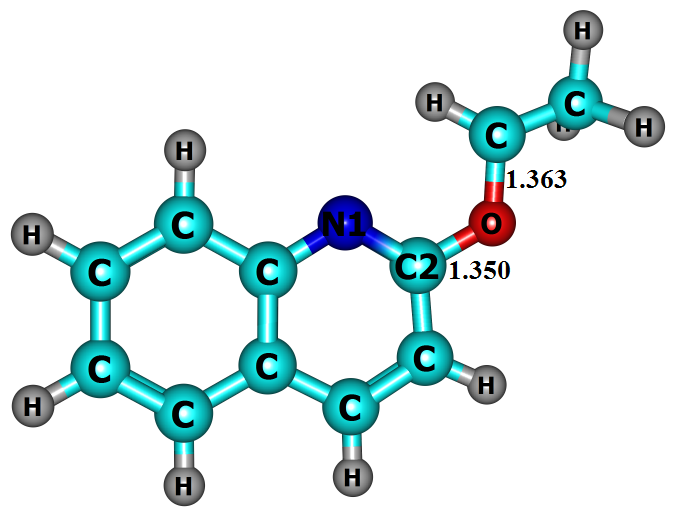**2-CH_3_^•^CHOQ** | 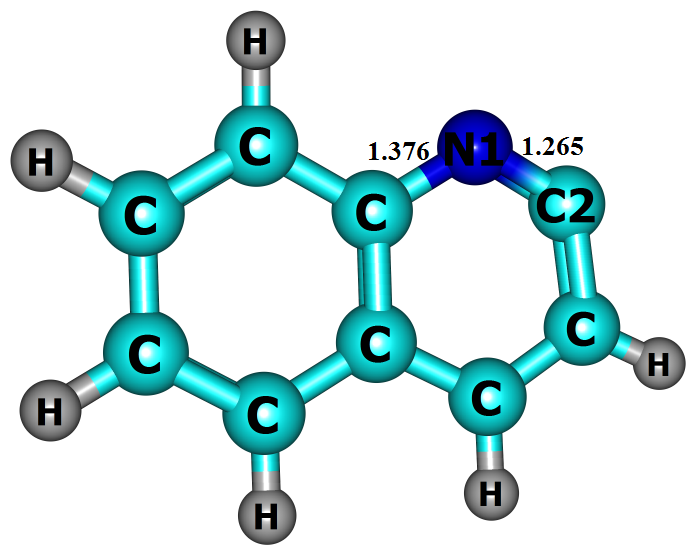**2-Q^•^** | 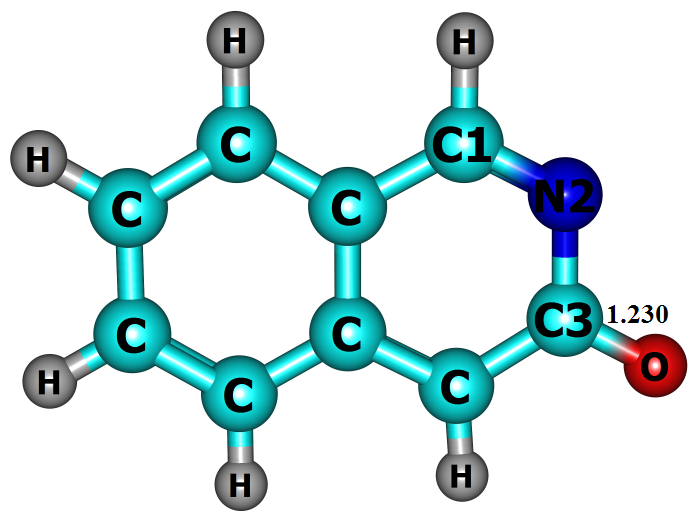**3-^•^OisoQ** | 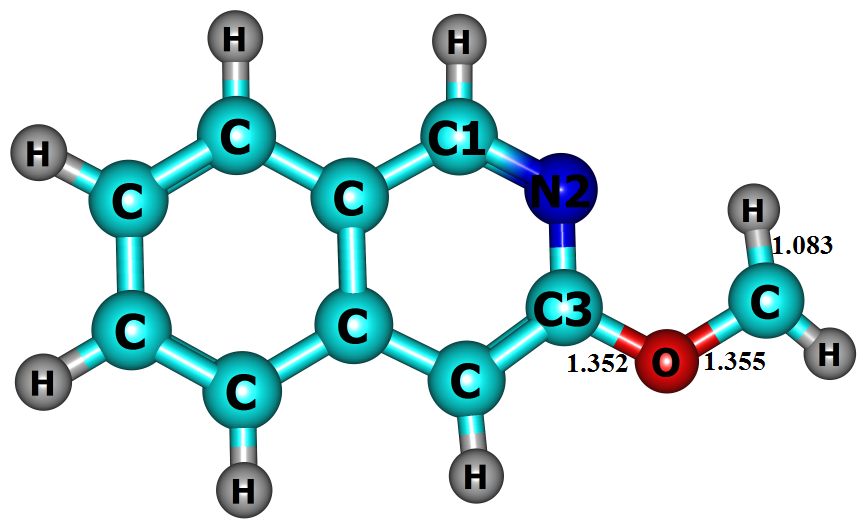**3-^•^CH_2_OisoQ** |
| 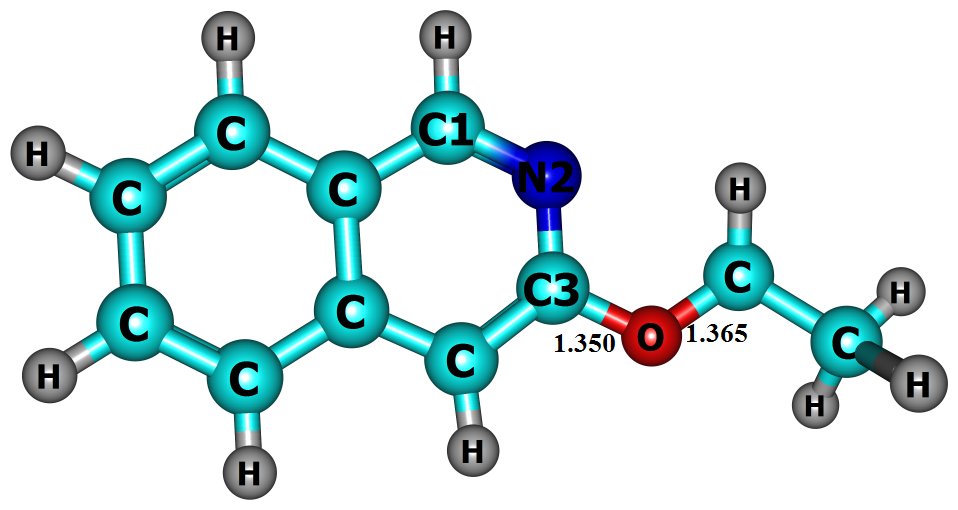**3-CH_3_^•^CHOisoQ** | 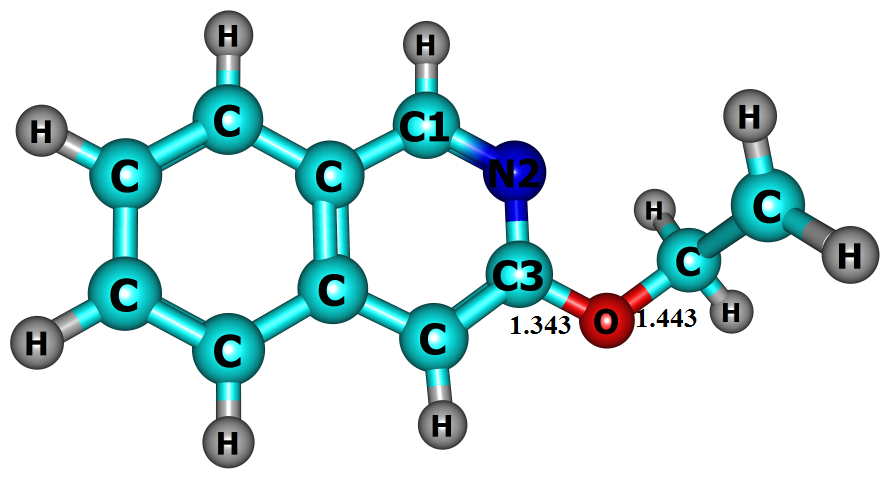**3-^•^CH_2_CH_2_OisoQ** | 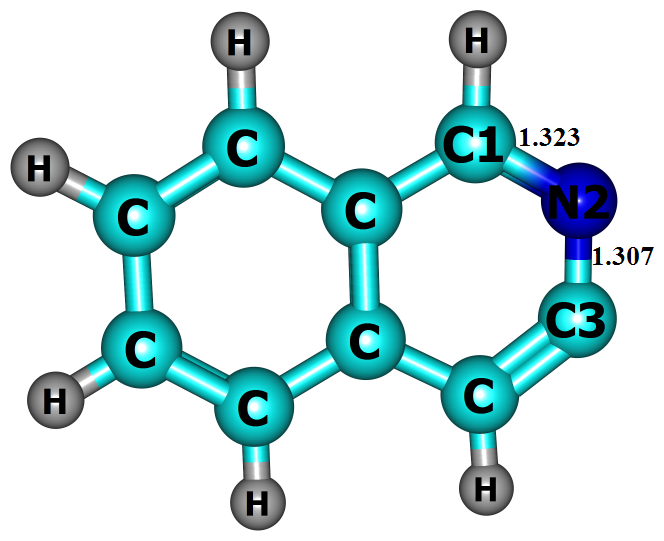**3-isoQ^•^** | 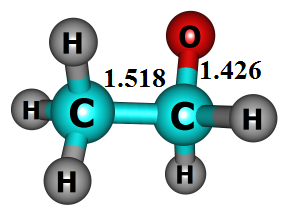**^•^OC_2_H_5_** |
| 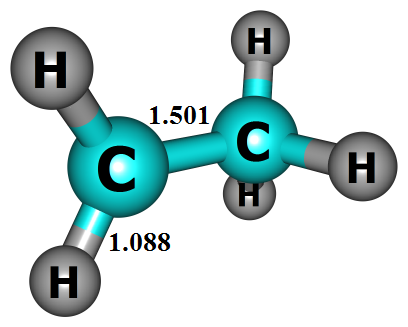**^•^C_2_H_5_** | 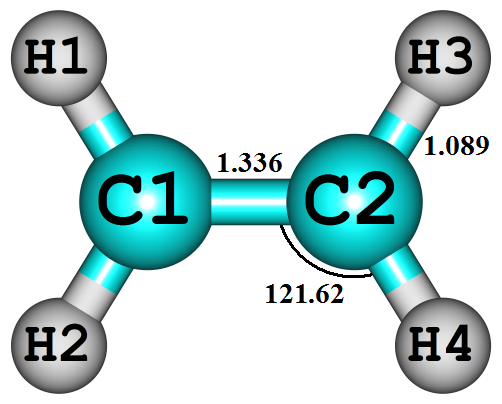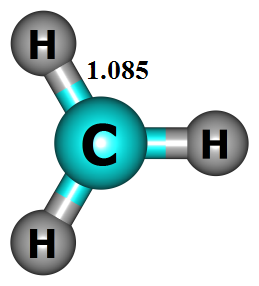**C_2_H_4_**, **^•^CH_3_** |  |  |

**Figure S1.** Optimized structures of **1-EisoQ**,**2-EQ**, **3-EQ, 3-EisoQ**,**4-EQ, 4-EisoQ**,**5-EQ**,**5-EisoQ, 8-EQ** and **8-EisoQ** transition states (TSs) and products for unimolecular decomposition reaction at BMK/6-31+G(d,p). Bond lengths are given in Ångstroms and angles in degrees.

Table S1. Optimized geometries of Reactants and different transition states at BMK/6-31+G(d,p) level.

| **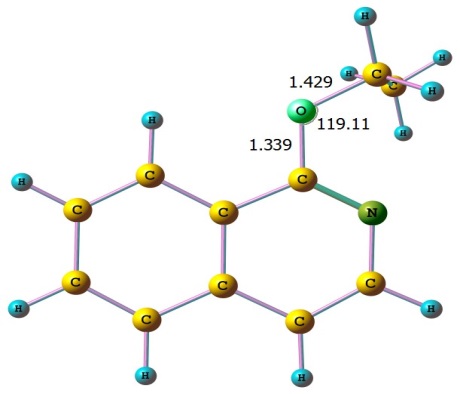**  **1-EisoQ**  1 -0.350277000 -2.426655000 -0.200077000  6 -1.061427000 -1.612217000 -0.091947000  6 -2.416177000 -1.859346000 0.040197000  6 -3.330399000 -0.779816000 0.178269000  6 -2.881266000 0.527546000 0.181423000  6 -1.491964000 0.813875000 0.046975000  6 -0.583123000 -0.274896000 -0.089335000  6 0.817815000 0.057723000 -0.221965000  7 1.282629000 1.280685000 -0.222831000  6 0.396245000 2.309866000 -0.096294000  6 -0.960103000 2.143038000 0.039579000  1 -2.785904000 -2.882510000 0.038703000  1 -4.393899000 -0.988309000 0.280172000  1 -3.580884000 1.355378000 0.286955000  1 -1.620355000 3.001338000 0.141309000  1 0.838595000 3.304507000 -0.106743000  8 1.647610000 -0.984054000 -0.355389000  6 3.051126000 -0.743799000 -0.476995000  1 3.214521000 0.110586000 -1.142765000  1 3.438440000 -1.654364000 -0.945883000  6 3.703635000 -0.508432000 0.887959000  1 4.789985000 -0.410622000 0.764405000  1 3.505073000 -1.353889000 1.557642000  1 3.319247000 0.410928000 1.342020000 | **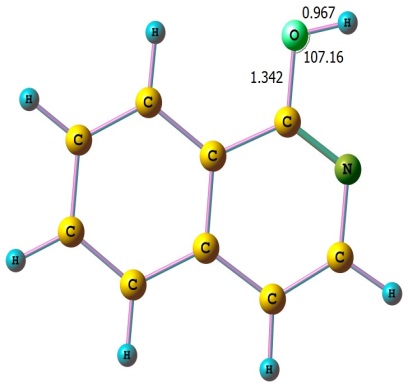**  **1-isoQ_enol_ (P1)**  1 0.734226000 -2.488099000 -0.000189000  6 1.035287000 -1.443864000 -0.000113000  6 2.370233000 -1.083115000 -0.000094000  6 2.742030000 0.289215000 -0.000009000  6 1.779645000 1.281598000 0.000060000  6 0.395726000 0.943056000 0.000046000  6 0.034015000 -0.435573000 -0.000057000  6 -1.374048000 -0.730657000 -0.000101000  7 -2.321100000 0.170965000 0.000027000  6 -1.957959000 1.485946000 0.000142000  6 -0.652964000 1.916631000 0.000126000  1 3.141996000 -1.849902000 -0.000150000  1 3.796591000 0.558230000 0.000005000  1 2.066423000 2.331899000 0.000132000  1 -0.416318000 2.978133000 0.000191000  1 -2.784944000 2.193326000 0.000205000  8 -1.728363000 -2.025385000 -0.000048000  1 -2.695163000 -2.056683000 0.000006000 |
| --- | --- |
| **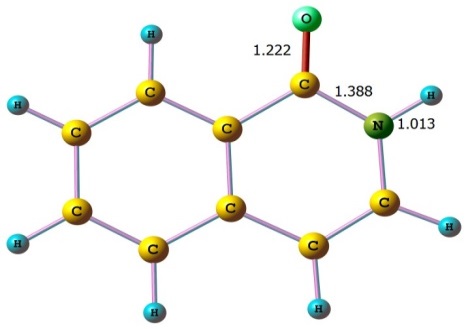**  **1-isoQ_keto_ (P2)**  1 0.837624000 -2.470323000 -0.000116000  6 1.108059000 -1.416915000 -0.000047000  6 2.435084000 -1.000553000 0.000022000  6 2.738645000 0.378520000 0.000106000  6 1.721719000 1.326873000 0.000124000  6 0.364300000 0.924722000 0.000056000  6 0.070467000 -0.464520000 -0.000034000  6 -1.334151000 -0.939208000 -0.000118000  7 -2.280196000 0.076221000 -0.000079000  6 -2.004559000 1.424549000 0.000024000  6 -0.727307000 1.880279000 0.000082000  1 3.239193000 -1.733173000 0.000012000  1 3.777414000 0.703372000 0.000158000  1 1.960082000 2.389428000 0.000190000  1 -0.526438000 2.947480000 0.000160000  1 -2.870269000 2.080329000 0.000047000  8 -1.681209000 -2.110737000 -0.000138000  1 -3.240111000 -0.247251000 -0.000088000 | 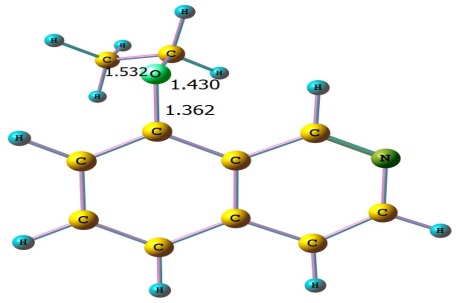  **8-EisoQ**  8 -1.837623000 -0.592905000 -0.868754000  6 -0.868516000 0.290517000 -0.498960000  6 -1.144356000 1.642954000 -0.435230000  6 -0.122763000 2.568126000 -0.079812000  6 1.155965000 2.139148000 0.211187000  6 1.472730000 0.750256000 0.136355000  6 0.458047000 -0.185294000 -0.233183000  6 0.826737000 -1.561776000 -0.347412000  7 2.034140000 -2.021158000 -0.102656000  6 2.991257000 -1.130536000 0.277091000  6 2.771206000 0.225237000 0.399592000  1 -2.150485000 1.984941000 -0.666182000  1 -0.365052000 3.628147000 -0.035531000  1 1.934186000 2.847635000 0.489421000  1 3.581033000 0.892616000 0.689188000  1 3.973735000 -1.554633000 0.477365000  6 -2.486826000 -1.307921000 0.185199000  1 -1.732731000 -1.723600000 0.870686000  1 -2.995460000 -2.139603000 -0.314276000  6 -3.491542000 -0.441329000 0.950774000  1 -2.989150000 0.387727000 1.463472000  1 -4.002977000 -1.051009000 1.706841000  1 -4.243193000 -0.030677000 0.265664000  1 0.080459000 -2.286487000 -0.671627000 |
| **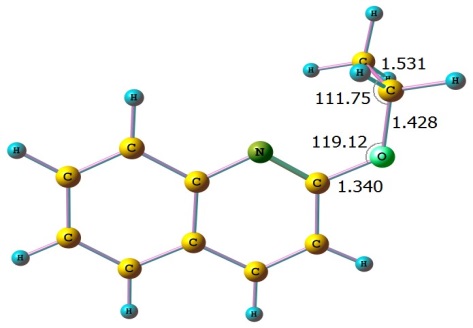**  **2-EQ**  1 -0.927430000 -2.558962000 -0.348058000  6 -1.489628000 -1.640052000 -0.197180000  6 -2.868075000 -1.640870000 -0.081821000  6 -3.582077000 -0.427608000 0.114491000  6 -2.896836000 0.771145000 0.193134000  6 -1.479913000 0.800650000 0.078431000  6 -0.762266000 -0.419347000 -0.120734000  7 0.601225000 -0.457137000 -0.237623000  6 1.261853000 0.668418000 -0.165671000  6 0.650808000 1.954601000 0.034637000  6 -0.711309000 2.006596000 0.153745000  1 -3.415452000 -2.579730000 -0.141701000  1 -4.666338000 -0.445053000 0.200850000  1 -3.433322000 1.707640000 0.343525000  1 -1.221521000 2.957294000 0.305410000  1 1.284176000 2.835681000 0.082720000  8 2.595897000 0.691886000 -0.285943000  6 3.297639000 -0.536053000 -0.484149000  1 4.251218000 -0.231626000 -0.927789000  1 2.744871000 -1.158211000 -1.196904000  6 3.522754000 -1.280390000 0.835138000  1 4.138772000 -2.170874000 0.654995000  1 2.567004000 -1.597725000 1.264764000  1 4.044577000 -0.636090000 1.552966000 | 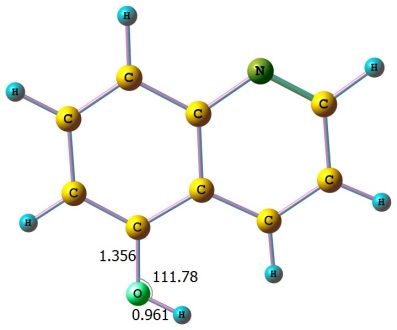  **5-Q_enol_ (P11)**  8 1.808664000 2.067910000 0.003442000  6 1.368921000 0.785393000 0.003404000  6 2.320135000 -0.217245000 -0.006472000  6 1.923021000 -1.579676000 -0.010370000  6 0.590323000 -1.938514000 0.000316000  6 -0.411000000 -0.926093000 0.005824000  6 -0.028761000 0.459266000 -0.001759000  6 -1.073541000 1.426514000 -0.018218000  6 -2.386030000 1.008372000 -0.011520000  6 -2.653262000 -0.391511000 0.007780000  7 -1.719385000 -1.314408000 0.013418000  1 3.370675000 0.061857000 -0.005773000  1 2.692899000 -2.348602000 -0.016283000  1 0.265909000 -2.975449000 0.003855000  1 -3.685003000 -0.744748000 0.015754000  1 -3.208370000 1.719425000 -0.025305000  1 1.089562000 2.691401000 0.136790000  1 -0.858129000 2.494659000 -0.044412000 |
| **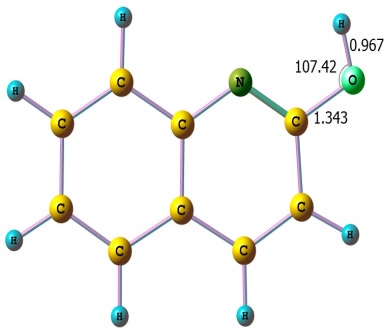**  **2-Q_enol_ (P3)**  1 1.084192000 -2.576859000 -0.000001000  6 1.317101000 -1.514432000 0.000000000  6 2.620752000 -1.054672000 0.000001000  6 2.901689000 0.339795000 0.000003000  6 1.864405000 1.253085000 0.000001000  6 0.512556000 0.809122000 0.000000000  6 0.231850000 -0.593625000 0.000000000  7 -1.046455000 -1.078343000 -0.000008000  6 -2.034339000 -0.223224000 -0.000016000  6 -1.881732000 1.200837000 -0.000002000  6 -0.605406000 1.701321000 0.000000000  1 3.445078000 -1.765363000 0.000002000  1 3.933727000 0.683424000 0.000005000  1 2.066699000 2.323739000 0.000003000  1 -0.431493000 2.776808000 0.000005000  1 -2.766242000 1.830883000 0.000004000  8 -3.291348000 -0.696419000 0.000013000  1 -3.237251000 -1.662111000 0.000017000 | 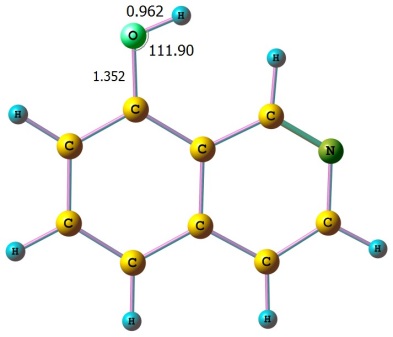  **8-isoQ_enol_ (P12)**  8 -1.777196000 -2.075108000 0.000036000  6 -1.360458000 -0.788829000 0.000025000  6 -2.329441000 0.198834000 -0.000032000  6 -1.954461000 1.566556000 -0.000053000  6 -0.627158000 1.950368000 -0.000002000  6 0.389260000 0.953652000 0.000027000  6 0.029969000 -0.432628000 -0.000006000  6 1.092139000 -1.387212000 -0.000103000  7 2.369771000 -1.081181000 -0.000055000  6 2.706204000 0.237478000 0.000023000  6 1.781600000 1.259489000 0.000060000  1 -3.375464000 -0.096437000 -0.000032000  1 -2.737940000 2.321889000 -0.000076000  1 -0.344823000 3.000891000 0.000037000  1 2.106964000 2.298110000 0.000103000  1 3.773916000 0.448984000 0.000112000  1 -1.038863000 -2.691292000 0.000549000  1 0.879452000 -2.459263000 -0.000241000 |
| **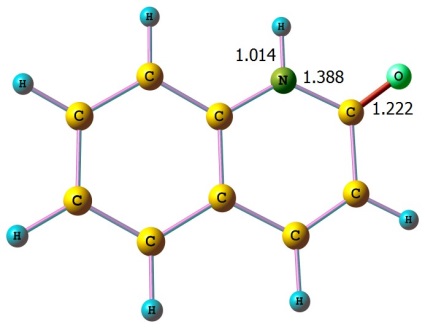**  **2-Q_keto_ (P4)**  1 1.153943000 -2.564001000 -0.000121000  6 1.340482000 -1.491238000 -0.000053000  6 2.641793000 -0.996933000 0.000016000  6 2.888260000 0.391908000 0.000106000  6 1.816168000 1.278103000 0.000128000  6 0.485572000 0.805932000 0.000058000  6 0.253904000 -0.592718000 -0.000034000  7 -1.049367000 -1.045868000 -0.000105000  6 -2.193072000 -0.258608000 -0.000109000  6 -1.933858000 1.192252000 0.000023000  6 -0.669442000 1.683039000 0.000088000  1 3.476935000 -1.694369000 -0.000001000  1 3.909515000 0.764639000 0.000159000  1 1.988847000 2.353578000 0.000198000  1 -0.497202000 2.759348000 0.000168000  1 -2.812965000 1.830136000 0.000055000  1 -1.228955000 -2.044060000 -0.000166000  8 -3.302924000 -0.769326000 -0.000112000 | 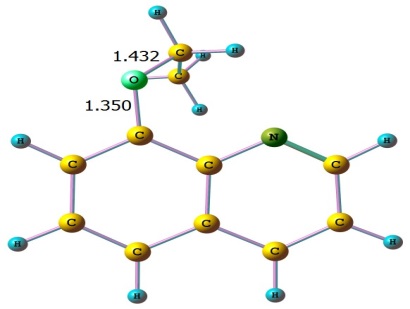  **8-EQ**  8 -2.087382000 0.200165000 -0.805318000  6 -0.892588000 0.686914000 -0.409278000  6 -0.802355000 2.061855000 -0.247397000  6 0.421679000 2.689913000 0.097326000  6 1.565291000 1.943817000 0.294179000  6 1.517438000 0.530302000 0.130561000  6 0.290697000 -0.121435000 -0.236134000  7 0.220988000 -1.466324000 -0.439369000  6 1.307556000 -2.189196000 -0.286829000  6 2.572262000 -1.651439000 0.088449000  6 2.668526000 -0.294087000 0.295290000  1 -1.704401000 2.650135000 -0.397439000  1 0.445682000 3.771729000 0.212362000  1 2.508087000 2.415687000 0.565537000  1 3.613896000 0.167543000 0.578807000  1 3.431085000 -2.308601000 0.202457000  1 1.206036000 -3.260555000 -0.465208000  6 -2.662778000 -0.971935000 -0.217507000  1 -2.113874000 -1.858181000 -0.545955000  1 -3.676397000 -0.991680000 -0.632001000  6 -2.713546000 -0.895813000 1.311122000  1 -1.704464000 -0.910077000 1.740423000  1 -3.261032000 -1.763603000 1.700610000  1 -3.225560000 0.017166000 1.639841000 |
| **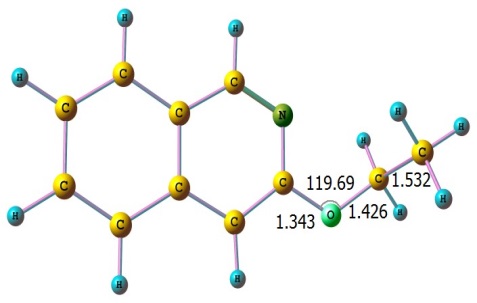**  **3-EisoQ**  1 -2.625321000 2.454065000 -0.060900000  6 -2.556988000 1.368174000 -0.003779000  6 -3.687141000 0.594687000 0.141789000  6 -3.573830000 -0.828481000 0.217204000  6 -2.346221000 -1.449628000 0.145580000  6 -1.152283000 -0.673304000 -0.006638000  6 -1.271166000 0.751914000 -0.080145000  6 -0.070065000 1.495923000 -0.227362000  7 1.130563000 0.956842000 -0.299904000  6 1.233674000 -0.389210000 -0.234298000  6 0.142058000 -1.238530000 -0.087672000  1 -4.669160000 1.058990000 0.198657000  1 -4.475012000 -1.427780000 0.333396000  1 -2.268574000 -2.534222000 0.201684000  1 0.297734000 -2.313486000 -0.041919000  1 -0.116009000 2.585560000 -0.282927000  8 2.449837000 -0.951327000 -0.324169000  6 3.606581000 -0.121719000 -0.416496000  1 3.417045000 0.700674000 -1.114870000  1 4.375936000 -0.780049000 -0.833625000  6 4.030595000 0.411929000 0.955105000  1 4.207504000 -0.418338000 1.649386000  1 4.958086000 0.990968000 0.857403000  1 3.253847000 1.065814000 1.366679000 | 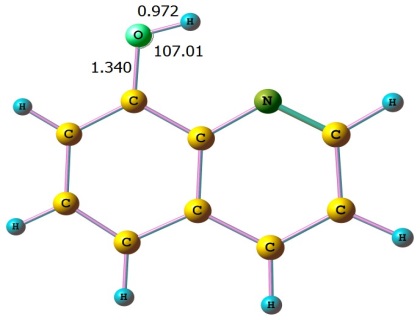  **8-Q_enol_ (P13)**  8 -1.841603000 1.934232000 0.000000000  6 -1.415335000 0.663582000 0.000000000  6 -2.303379000 -0.396181000 0.000000000  6 -1.812127000 -1.730658000 0.000000000  6 -0.458095000 -2.009743000 0.000000000  6 0.478603000 -0.934898000 0.000000000  6 0.000000000 0.409831000 0.000000000  7 0.812644000 1.498377000 0.000000000  6 2.113984000 1.305845000 0.000000000  6 2.710009000 0.011126000 0.000000000  6 1.893721000 -1.101054000 0.000000000  1 -3.371430000 -0.193541000 0.000000000  1 -2.529672000 -2.548921000 0.000000000  1 -0.096501000 -3.036150000 0.000000000  1 2.313226000 -2.106489000 0.000000000  1 3.793446000 -0.081289000 0.000000000  1 2.741424000 2.197031000 0.000000000  1 -1.050462000 2.499756000 0.000000000 |
| **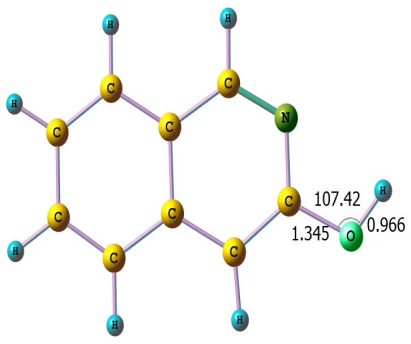**  **3-isoQ_enol_ (P5)**  1 -1.946205000 2.379028000 -0.000276000  6 -1.813843000 1.297547000 -0.000216000  6 -2.901972000 0.452104000 -0.000195000  6 -2.706004000 -0.962881000 -0.000010000  6 -1.438887000 -1.505842000 0.000148000  6 -0.289655000 -0.654558000 0.000140000  6 -0.490846000 0.763051000 0.000053000  6 0.668859000 1.587336000 0.000477000  7 1.899806000 1.121402000 0.000034000  6 2.076593000 -0.219690000 -0.000277000  6 1.042697000 -1.140929000 0.000161000  1 -3.912735000 0.853894000 -0.000292000  1 -3.574172000 -1.619376000 -0.000087000  1 -1.297024000 -2.585445000 0.000186000  1 1.261083000 -2.205666000 0.000354000  1 0.559242000 2.673477000 0.000138000  8 3.351358000 -0.647186000 -0.000264000  1 3.918652000 0.134927000 0.000164000 | 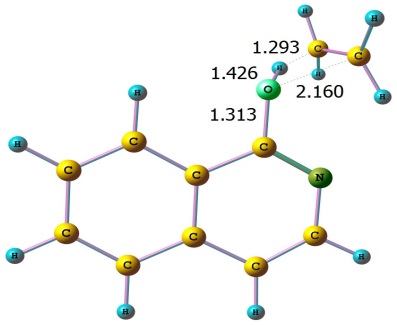  **TS1_enol_**  1 -0.295832000 -2.380881000 -0.387262000  6 -1.048993000 -1.618806000 -0.203655000  6 -2.385071000 -1.946405000 -0.049447000  6 -3.346710000 -0.925388000 0.181015000  6 -2.962590000 0.401522000 0.248857000  6 -1.593950000 0.766608000 0.091021000  6 -0.634902000 -0.263005000 -0.133679000  6 0.760983000 0.120160000 -0.288932000  7 1.150177000 1.388207000 -0.239797000  6 0.215490000 2.355359000 -0.028639000  6 -1.128455000 2.118048000 0.142160000  1 -2.704305000 -2.984952000 -0.107219000  1 -4.395055000 -1.192616000 0.301827000  1 -3.700266000 1.183715000 0.422844000  1 -1.824499000 2.937651000 0.306020000  1 0.603605000 3.373171000 -0.002470000  8 1.662246000 -0.816479000 -0.474405000  6 3.730241000 -0.215570000 -0.306153000  1 3.429162000 0.831617000 -0.311898000  1 3.937072000 -0.678804000 -1.267927000  6 3.857429000 -0.937903000 0.891797000  1 4.337722000 -1.914612000 0.878509000  1 2.623371000 -1.095843000 0.541346000  1 3.858972000 -0.391782000 1.833978000 |
| 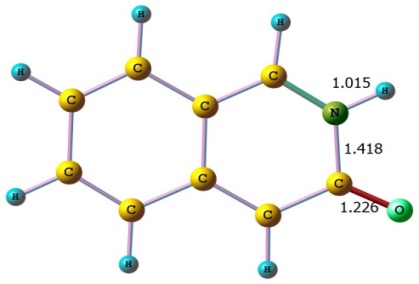  **3-isoQ_keto_ (P6)**  1 1.945516000 2.386690000 0.000912000  6 1.812760000 1.305335000 0.000545000  6 2.889376000 0.463678000 0.000486000  6 2.686344000 -0.966511000 -0.000099000  6 1.432177000 -1.511393000 -0.000368000  6 0.256328000 -0.666907000 -0.000193000  6 0.474025000 0.771331000 0.000133000  6 -0.648121000 1.584886000 -0.000487000  7 -1.881506000 1.051339000 -0.000914000  6 -2.199337000 -0.330220000 0.000171000  6 -1.035135000 -1.179384000 -0.000407000  1 3.902073000 0.859501000 0.000798000  1 3.556963000 -1.619863000 -0.000198000  1 1.293639000 -2.591118000 -0.000620000  1 -1.216941000 -2.251001000 -0.000843000  1 -0.583449000 2.671061000 -0.000703000  8 -3.379772000 -0.661486000 0.001120000  1 -2.699577000 1.652353000 -0.000591000 | 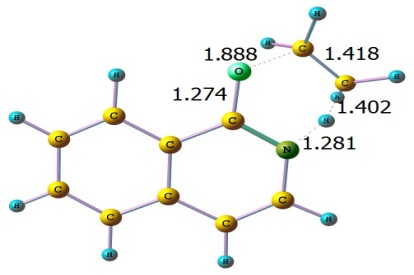  **TS2_keto_**  1 -0.687580000 -2.478630000 -0.243874000  6 -1.288689000 -1.583240000 -0.104953000  6 -2.658734000 -1.651552000 0.097605000  6 -3.411258000 -0.461420000 0.261484000  6 -2.791046000 0.777988000 0.227142000  6 -1.387831000 0.874828000 0.027045000  6 -0.643530000 -0.325031000 -0.143728000  6 0.795828000 -0.224313000 -0.382447000  7 1.385369000 0.987610000 -0.409691000  6 0.669139000 2.131708000 -0.176199000  6 -0.683647000 2.132100000 0.010121000  1 -3.160423000 -2.616496000 0.125742000  1 -4.487203000 -0.521647000 0.415123000  1 -3.372371000 1.689950000 0.355288000  1 -1.225265000 3.065210000 0.144887000  1 1.250938000 3.051470000 -0.178225000  8 1.522624000 -1.257493000 -0.551097000  6 3.204830000 -1.240811000 0.304945000  1 3.637276000 -1.776220000 -0.535698000  1 2.815397000 -1.881755000 1.092744000  6 3.641936000 0.073006000 0.610244000  1 4.482223000 0.458731000 0.029947000  1 3.618054000 0.376597000 1.657478000  1 2.568391000 0.759883000 0.025647000 |
| **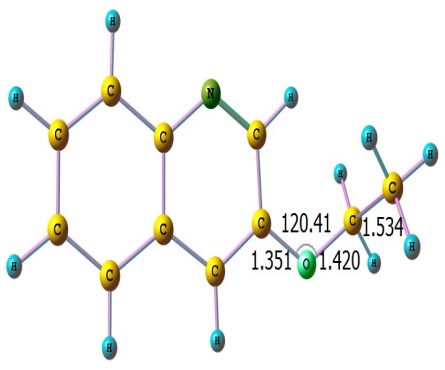**  **3-EQ**  1 -2.618696000 2.447438000 -0.072184000  6 -2.563175000 1.362760000 -0.010957000  6 -3.688080000 0.578485000 0.128588000  6 -3.571514000 -0.841795000 0.208068000  6 -2.335760000 -1.450233000 0.145360000  6 -1.150798000 -0.664333000 -0.000404000  6 -1.270013000 0.761213000 -0.077941000  7 -0.181438000 1.564223000 -0.213150000  6 1.013301000 1.020177000 -0.277838000  6 1.243023000 -0.394310000 -0.217894000  6 0.148356000 -1.227803000 -0.075462000  1 -4.672912000 1.038379000 0.177756000  1 -4.468594000 -1.447713000 0.319400000  1 -2.246209000 -2.534182000 0.203262000  1 0.296100000 -2.305440000 -0.029004000  8 2.461385000 -0.971224000 -0.307227000  6 3.628531000 -0.171321000 -0.421836000  1 3.481610000 0.604396000 -1.187296000  1 4.397248000 -0.859234000 -0.789203000  6 4.055561000 0.431578000 0.922140000  1 4.244841000 -0.368404000 1.647253000  1 4.976790000 1.013923000 0.793408000  1 3.283953000 1.093681000 1.332178000  1 1.848262000 1.710873000 -0.376650000 | 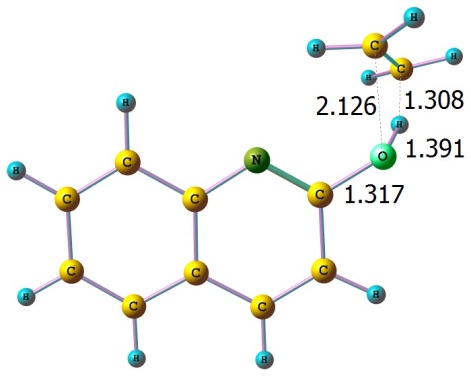  **TS3_enol_**  1 -1.037094000 -2.568254000 -0.393633000  6 -1.583144000 -1.647807000 -0.198350000  6 -2.950750000 -1.648395000 0.009295000  6 -3.649035000 -0.435233000 0.257473000  6 -2.955983000 0.761480000 0.293375000  6 -1.549839000 0.789877000 0.085028000  6 -0.844903000 -0.429329000 -0.164694000  7 0.505605000 -0.473884000 -0.370405000  6 1.194757000 0.655496000 -0.347589000  6 0.573221000 1.939763000 -0.105158000  6 -0.777145000 1.994688000 0.108470000  1 -3.501116000 -2.587073000 -0.019587000  1 -4.724919000 -0.451513000 0.417290000  1 -3.478174000 1.699367000 0.481400000  1 -1.275695000 2.946397000 0.292303000  1 1.202215000 2.825879000 -0.106980000  8 2.497228000 0.613854000 -0.540124000  6 3.493366000 -1.228947000 -0.177913000  1 3.950293000 -1.191648000 -1.163751000  1 2.539435000 -1.749645000 -0.100182000  6 4.132098000 -0.672156000 0.943310000  1 5.169525000 -0.353084000 0.860727000  1 3.768705000 -0.933591000 1.936075000  1 3.373901000 0.292904000 0.490684000 |
| 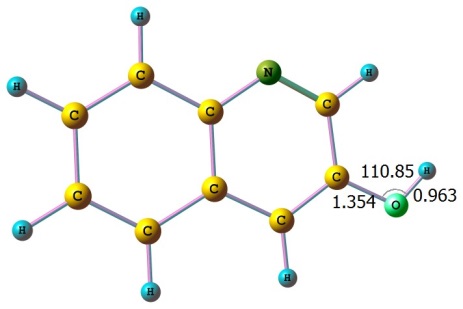  **3-Q_enol_ (P7)**  1 -1.925122000 2.377425000 -0.000011000  6 -1.809461000 1.295750000 -0.000008000  6 -2.893800000 0.443555000 -0.000025000  6 -2.698964000 -0.969193000 0.000006000  6 -1.426867000 -1.503238000 0.000007000  6 -0.284308000 -0.647310000 0.000001000  6 -0.482580000 0.772251000 0.000029000  7 0.563720000 1.645539000 0.000026000  6 1.782409000 1.165449000 -0.000046000  6 2.087600000 -0.232007000 -0.000007000  6 1.050121000 -1.138576000 0.000021000  1 -3.905174000 0.844589000 -0.000046000  1 -3.563993000 -1.629461000 0.000024000  1 -1.276886000 -2.582115000 0.000020000  1 1.256204000 -2.207386000 0.000054000  8 3.367146000 -0.674553000 -0.000071000  1 3.985268000 0.063865000 0.000408000  1 2.601608000 1.890637000 0.000066000 | 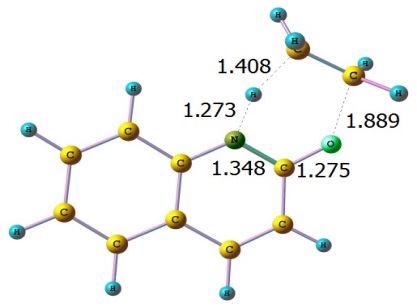  **TS4_keto_**  1 0.797872000 -2.482388000 -0.544283000  6 1.363571000 -1.582415000 -0.311984000  6 2.736291000 -1.624875000 -0.117945000  6 3.465278000 -0.444263000 0.168438000  6 2.800757000 0.768678000 0.265697000  6 1.396331000 0.839417000 0.084378000  6 0.672368000 -0.349046000 -0.213968000  7 -0.684745000 -0.310927000 -0.435097000  6 -1.389051000 0.830040000 -0.297989000  6 -0.706946000 2.068476000 0.043562000  6 0.647395000 2.065168000 0.202087000  1 3.260903000 -2.575533000 -0.191858000  1 4.542332000 -0.491604000 0.311938000  1 3.348626000 1.683453000 0.489746000  1 1.182261000 2.985530000 0.436427000  1 -1.313435000 2.964380000 0.144423000  8 -2.653317000 0.788073000 -0.454114000  6 -3.528451000 -0.706740000 0.299356000  1 -4.204036000 -0.746379000 -0.550625000  1 -3.877861000 -0.086622000 1.121553000  6 -2.641475000 -1.784820000 0.544828000  1 -2.358793000 -1.978600000 1.580213000  1 -1.494959000 -1.207887000 -0.034991000  1 -2.759570000 -2.670177000 -0.082709000 |
| **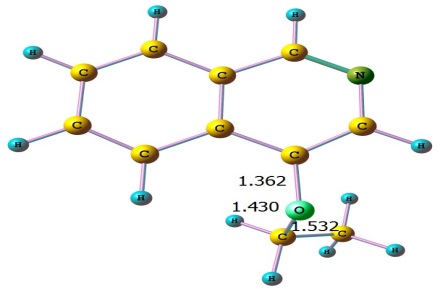**  **4-EisoQ**  8 -1.852659000 -0.605174000 -0.867984000  6 -0.872653000 0.264690000 -0.496374000  6 -1.131375000 1.620973000 -0.422982000  7 -0.195359000 2.548445000 -0.089931000  6 1.022334000 2.136753000 0.177519000  6 1.429064000 0.767631000 0.121661000  6 0.452411000 -0.210155000 -0.236746000  6 0.837731000 -1.579114000 -0.339454000  6 2.140753000 -1.949527000 -0.072141000  6 3.113777000 -0.977818000 0.304144000  6 2.765894000 0.354367000 0.394355000  1 -2.130978000 1.988524000 -0.648452000  1 3.504677000 1.106373000 0.668647000  1 4.133962000 -1.293298000 0.512245000  1 2.434897000 -2.994055000 -0.154407000  6 -2.506384000 -1.308735000 0.190893000  1 -1.753255000 -1.719761000 0.880824000  1 -3.017794000 -2.143365000 -0.301217000  6 -3.509424000 -0.431211000 0.945778000  1 -3.006170000 0.403541000 1.448217000  1 -4.023075000 -1.030185000 1.708975000  1 -4.258987000 -0.027170000 0.254657000  1 0.098831000 -2.315297000 -0.646538000  1 1.753918000 2.899841000 0.450526000 | 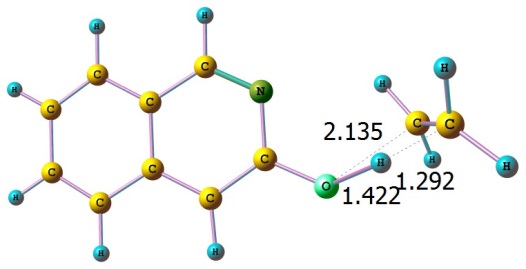  **TS5_enol_**  1 -2.674360000 2.472203000 0.038200000  6 -2.625115000 1.383563000 0.039178000  6 -3.775938000 0.624767000 0.087850000  6 -3.688747000 -0.800846000 0.086929000  6 -2.467055000 -1.438791000 0.038921000  6 -1.255070000 -0.678494000 -0.010534000  6 -1.349642000 0.749195000 -0.010202000  6 -0.127218000 1.479320000 -0.068518000  7 1.065024000 0.929554000 -0.113941000  6 1.166382000 -0.440501000 -0.103828000  6 0.035661000 -1.259683000 -0.066034000  1 -4.751342000 1.104589000 0.126876000  1 -4.603879000 -1.389180000 0.124705000  1 -2.408123000 -2.526172000 0.038119000  1 0.166780000 -2.339064000 -0.071426000  1 -0.163036000 2.571771000 -0.080729000  8 2.382467000 -0.949341000 -0.126338000  6 4.052384000 0.297873000 -0.588323000  1 3.398211000 1.123081000 -0.861617000  1 4.352102000 -0.389408000 -1.375484000  6 4.532650000 0.163638000 0.727778000  1 5.347537000 -0.529730000 0.927489000  1 4.411009000 1.002788000 1.411346000  1 3.420443000 -0.493277000 0.731505000 |
| 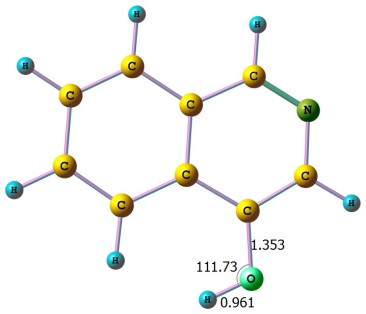  **4-isoQ_enol_ (P8)**  8 1.887960000 2.002094000 0.000001000  6 1.391437000 0.743417000 0.000010000  6 2.297309000 -0.304628000 -0.000010000  7 1.930715000 -1.608281000 -0.000022000  6 0.651435000 -1.905381000 0.000001000  6 -0.384063000 -0.921641000 0.000012000  6 -0.011145000 0.458320000 -0.000007000  6 -1.038375000 1.450233000 -0.000048000  6 -2.368041000 1.078826000 -0.000028000  6 -2.738400000 -0.296312000 0.000018000  6 -1.765276000 -1.273845000 0.000031000  1 3.363460000 -0.087031000 -0.000012000  1 0.384072000 -2.963299000 0.000009000  1 -3.142353000 1.843224000 -0.000061000  1 1.187808000 2.660931000 0.000373000  1 -0.793332000 2.511598000 -0.000120000  1 -2.036412000 -2.328611000 0.000052000  1 -3.791223000 -0.569538000 0.000033000 | 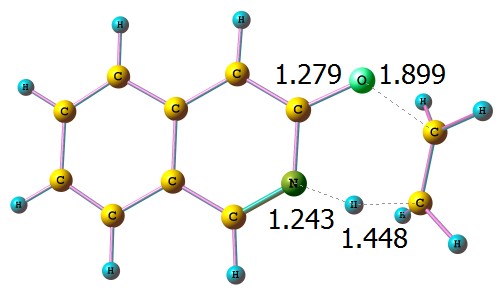  **TS6_keto_**  1 -2.407064000 2.524642000 -0.147622000  6 -2.417941000 1.439324000 -0.052891000  6 -3.597113000 0.759081000 0.124584000  6 -3.587112000 -0.670782000 0.248670000  6 -2.414544000 -1.383999000 0.192208000  6 -1.156738000 -0.711104000 0.006402000  6 -1.175902000 0.726376000 -0.113401000  6 0.058945000 1.375381000 -0.282323000  7 1.198489000 0.706924000 -0.396115000  6 1.265153000 -0.672022000 -0.295060000  6 0.073398000 -1.388022000 -0.051460000  1 -4.541407000 1.296429000 0.173276000  1 -4.530055000 -1.196355000 0.389043000  1 -2.418379000 -2.468843000 0.283373000  1 0.132200000 -2.468054000 0.057561000  1 0.119899000 2.463092000 -0.342600000  8 2.420159000 -1.202516000 -0.435815000  6 3.885870000 -0.284735000 0.349138000  1 3.842985000 -0.945581000 1.211945000  1 4.516212000 -0.643630000 -0.459608000  6 3.654074000 1.101748000 0.508456000  1 2.326775000 1.112497000 -0.070047000  1 4.185558000 1.777768000 -0.163681000  1 3.494037000 1.472206000 1.521750000 |
| 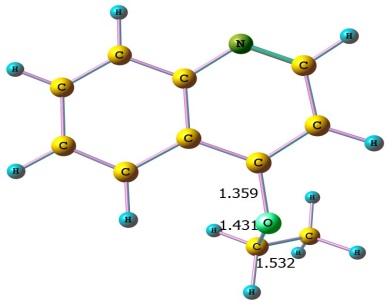  **4-EQ**  8 -1.869478000 -0.574395000 -0.876177000  6 -0.886292000 0.288319000 -0.508863000  6 -1.128539000 1.644492000 -0.436829000  6 -0.060398000 2.511125000 -0.069800000  7 1.157794000 2.108784000 0.215464000  6 1.423251000 0.772491000 0.129351000  6 0.435975000 -0.196477000 -0.244345000  6 0.805536000 -1.568314000 -0.361333000  6 2.099297000 -1.964544000 -0.087998000  6 3.077423000 -1.008146000 0.310263000  6 2.749366000 0.327896000 0.411992000  1 -2.118201000 2.035804000 -0.658078000  1 -0.243876000 3.584432000 -0.006799000  1 3.480247000 1.080769000 0.698404000  1 4.092018000 -1.337568000 0.525424000  1 2.379279000 -3.011654000 -0.183643000  6 -2.502179000 -1.306279000 0.177967000  1 -1.736224000 -1.736093000 0.840297000  1 -3.025538000 -2.125356000 -0.326896000  6 -3.485968000 -0.444283000 0.974806000  1 -2.967609000 0.375016000 1.487392000  1 -3.985599000 -1.060354000 1.733538000  1 -4.248502000 -0.020226000 0.310458000  1 0.060437000 -2.288785000 -0.690205000 | 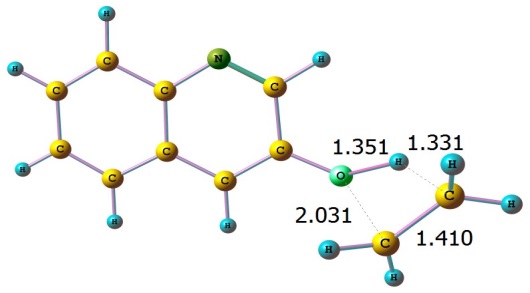  **TS7_enol_**  1 2.979924000 2.216536000 -0.435394000  6 2.763175000 1.159074000 -0.300023000  6 3.744350000 0.193212000 -0.401862000  6 3.419054000 -1.181601000 -0.214358000  6 2.123020000 -1.564511000 0.067761000  6 1.085967000 -0.588813000 0.176946000  6 1.417272000 0.792827000 -0.007450000  7 0.476803000 1.777918000 0.089414000  6 -0.760184000 1.443494000 0.354475000  6 -1.211684000 0.087918000 0.553531000  6 -0.266677000 -0.923504000 0.464005000  1 4.770714000 0.477443000 -0.624207000  1 4.201427000 -1.933975000 -0.293739000  1 1.874108000 -2.615153000 0.212916000  1 -0.558223000 -1.961032000 0.622865000  8 -2.501901000 -0.141991000 0.785981000  6 -3.715098000 -1.003792000 -0.595489000  1 -4.044399000 -1.778815000 0.091709000  1 -2.896104000 -1.265864000 -1.264340000  6 -4.431677000 0.201642000 -0.746604000  1 -4.258352000 0.800900000 -1.639315000  1 -3.493756000 0.514194000 0.145073000  1 -5.408768000 0.292786000 -0.275309000  1 -1.494090000 2.247802000 0.440412000 |
| 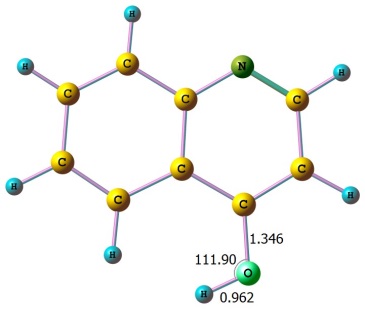  **4-Q_enol_ (P9)**  8 1.869150000 2.022448000 0.000035000  6 1.397891000 0.761194000 0.000014000  6 2.311079000 -0.275808000 -0.000018000  6 1.826396000 -1.608020000 -0.000031000  7 0.553258000 -1.943578000 -0.000006000  6 -0.361878000 -0.933451000 0.000006000  6 -0.003777000 0.455825000 -0.000009000  6 -1.036460000 1.439838000 -0.000050000  6 -2.365485000 1.065840000 -0.000030000  6 -2.722504000 -0.311415000 0.000019000  6 -1.745122000 -1.284561000 0.000029000  1 3.376278000 -0.062449000 -0.000011000  1 -3.142863000 1.826838000 -0.000064000  1 1.158675000 2.670959000 0.000344000  1 -0.797166000 2.503074000 -0.000125000  1 -1.990099000 -2.344118000 0.000051000  1 -3.773090000 -0.594029000 0.000036000  1 2.541415000 -2.431455000 -0.000049000 | 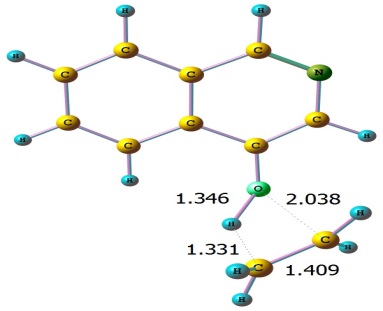  **TS8_enol_**  8 1.860826000 -0.225309000 -0.755907000  6 0.773741000 0.474556000 -0.440392000  6 0.823022000 1.859431000 -0.280997000  7 -0.241174000 2.620360000 0.072052000  6 -1.400449000 2.037319000 0.273301000  6 -1.603164000 0.628897000 0.133712000  6 -0.489920000 -0.185050000 -0.235358000  6 -0.670578000 -1.589599000 -0.391055000  6 -1.911200000 -2.157776000 -0.173752000  6 -3.021661000 -1.350210000 0.205517000  6 -2.870459000 0.014037000 0.353759000  1 1.760049000 2.385550000 -0.459857000  1 -3.715298000 0.639741000 0.639152000  1 -3.991405000 -1.814360000 0.373323000  1 -2.046243000 -3.230450000 -0.297773000  6 3.583530000 -0.097416000 0.324761000  1 3.224522000 0.725003000 0.942716000  1 4.176734000 0.173383000 -0.544823000  6 3.442635000 -1.438397000 0.734565000  1 3.108978000 -1.639111000 1.751605000  1 4.055943000 -2.200228000 0.256565000  1 2.358377000 -1.227671000 -0.007546000  1 0.175399000 -2.201983000 -0.694027000  1 -2.238434000 2.675318000 0.559195000 |
| 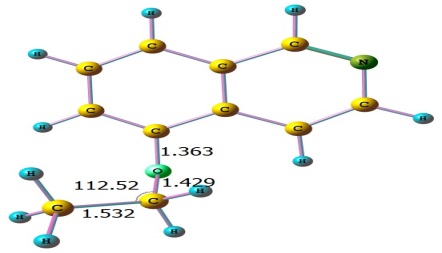  **5-EisoQ**  8 -1.853284000 -0.592973000 -0.871342000  6 -0.877387000 0.283839000 -0.500523000  6 -1.140484000 1.638619000 -0.435461000  6 -0.116583000 2.563083000 -0.081782000  6 1.158088000 2.121801000 0.206862000  6 1.458639000 0.730738000 0.129780000  6 0.445434000 -0.205186000 -0.237325000  6 0.818484000 -1.575253000 -0.342269000  6 2.120565000 -1.937647000 -0.065795000  7 3.090217000 -1.056417000 0.307064000  6 2.765926000 0.213133000 0.392863000  1 -2.145277000 1.985761000 -0.666142000  1 -0.354048000 3.623902000 -0.038759000  1 1.945229000 2.821391000 0.483817000  1 2.436652000 -2.976537000 -0.140560000  6 -2.502873000 -1.301516000 0.186218000  1 -1.749272000 -1.710287000 0.876519000  1 -3.010437000 -2.137764000 -0.307072000  6 -3.510377000 -0.430876000 0.943675000  1 -3.010288000 0.403700000 1.449658000  1 -4.021170000 -1.034864000 1.704807000  1 -4.262290000 -0.028134000 0.254201000  1 0.089074000 -2.318109000 -0.654424000  1 3.559983000 0.905231000 0.681785000 | 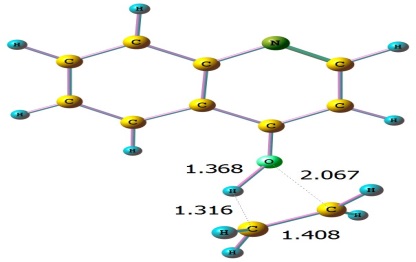  **TS9_enol_**  8 1.867388000 -0.159911000 -0.748650000  6 0.775801000 0.517931000 -0.440659000  6 0.786410000 1.902460000 -0.280418000  6 -0.411541000 2.565525000 0.088302000  7 -1.569672000 1.975053000 0.299019000  6 -1.614836000 0.619334000 0.136896000  6 -0.478504000 -0.168467000 -0.235873000  6 -0.618632000 -1.575835000 -0.396525000  6 -1.839083000 -2.187365000 -0.181095000  6 -2.968617000 -1.411321000 0.202086000  6 -2.859588000 -0.043587000 0.355445000  1 1.699401000 2.467116000 -0.454124000  1 -0.395078000 3.649350000 0.215230000  1 -3.709182000 0.571915000 0.642781000  1 -3.925444000 -1.902216000 0.369852000  1 -1.940427000 -3.262949000 -0.310472000  6 3.615139000 -0.080648000 0.351712000  1 3.252480000 0.716757000 0.999178000  1 4.204321000 0.222388000 -0.509891000  6 3.456153000 -1.435723000 0.697913000  1 3.114497000 -1.679898000 1.702847000  1 4.061714000 -2.182153000 0.186572000  1 2.384144000 -1.200831000 -0.027653000  1 0.245957000 -2.159384000 -0.704958000 |
| 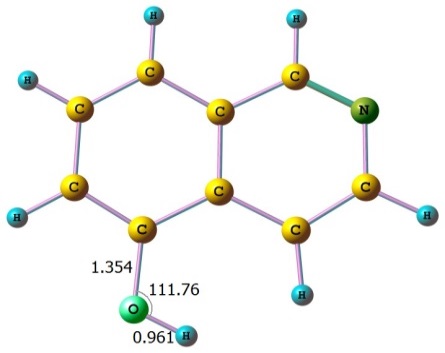  **5-isoQ_enol_ (P10)**  8 1.803982000 2.075706000 0.000018000  6 1.371165000 0.793207000 0.000007000  6 2.327281000 -0.206730000 -0.000025000  6 1.945197000 -1.573226000 -0.000032000  6 0.613244000 -1.937756000 0.000000000  6 -0.386474000 -0.925124000 0.000019000  6 -0.023513000 0.457471000 -0.000009000  6 -1.085713000 1.409041000 -0.000053000  6 -2.392804000 0.968034000 -0.000043000  7 -2.746255000 -0.346164000 0.000019000  6 -1.782743000 -1.237505000 0.000055000  1 3.376022000 0.079937000 -0.000026000  1 2.721877000 -2.335027000 -0.000053000  1 0.313577000 -2.983811000 0.000016000  1 -3.218371000 1.677302000 -0.000074000  1 1.071927000 2.698945000 0.000411000  1 -0.902361000 2.482671000 -0.000123000  1 -2.084588000 -2.286986000 0.000059000 | 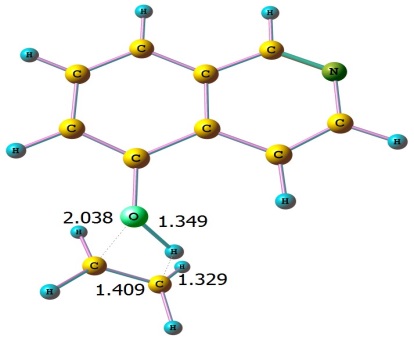  **TS10_enol_**  8 1.868656000 -0.162800000 -0.759855000  6 0.772159000 0.522592000 -0.443345000  6 0.797972000 1.907284000 -0.290793000  6 -0.369693000 2.627771000 0.079582000  6 -1.569865000 1.980625000 0.296188000  6 -1.634298000 0.565921000 0.136895000  6 -0.474145000 -0.177900000 -0.235649000  6 -0.613956000 -1.584785000 -0.391826000  6 -1.844682000 -2.169294000 -0.167652000  7 -2.954236000 -1.472999000 0.201003000  6 -2.843042000 -0.171403000 0.341015000  1 1.730250000 2.436320000 -0.478462000  1 -0.305193000 3.708714000 0.189963000  1 -3.745452000 0.370309000 0.633109000  1 -1.980659000 -3.243091000 -0.283773000  6 3.575906000 -0.069784000 0.348621000  1 3.206092000 0.732480000 0.986270000  1 4.180605000 0.227886000 -0.504062000  6 3.433158000 -1.422267000 0.716091000  1 3.083503000 -1.655450000 1.720706000  1 4.052429000 -2.168919000 0.222219000  1 2.360439000 -1.187913000 -0.033319000  1 0.236220000 -2.189253000 -0.697547000  1 -2.464927000 2.529757000 0.581953000 |
| 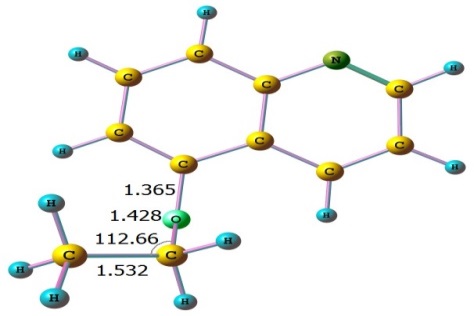  **5-EQ**  8 -1.843030000 -0.606463000 -0.865792000  6 -0.870169000 0.278002000 -0.498040000  6 -1.137529000 1.630961000 -0.434222000  6 -0.108367000 2.550071000 -0.081834000  6 1.167106000 2.112239000 0.206430000  6 1.476532000 0.721413000 0.133046000  6 0.452601000 -0.210706000 -0.235726000  6 0.810983000 -1.583239000 -0.349028000  6 2.107557000 -1.958739000 -0.073963000  6 3.043553000 -0.954040000 0.311130000  7 2.753673000 0.324189000 0.406109000  1 -2.141996000 1.978991000 -0.663790000  1 -0.343216000 3.611650000 -0.038592000  1 1.965800000 2.796066000 0.483257000  1 4.072159000 -1.235252000 0.540606000  1 2.427801000 -2.995074000 -0.150837000  6 -2.502315000 -1.298790000 0.195397000  1 -1.754721000 -1.694816000 0.900307000  1 -3.002903000 -2.144441000 -0.289381000  6 -3.520672000 -0.421027000 0.929939000  1 -3.028796000 0.421015000 1.431222000  1 -4.039650000 -1.016966000 1.691959000  1 -4.264616000 -0.029008000 0.225762000  1 0.062985000 -2.306651000 -0.665718000 | 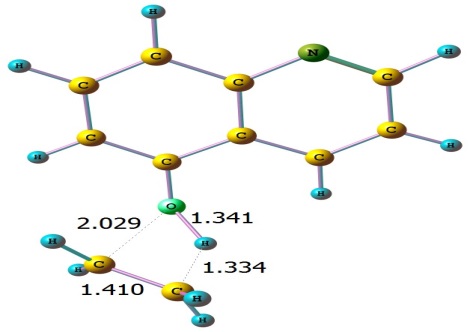**TS11_enol_**  8 -1.862576000 -0.191702000 -0.764282000  6 -0.771033000 0.505658000 -0.445447000  6 -0.812298000 1.888878000 -0.294148000  6 0.352442000 2.612073000 0.079017000  6 1.557583000 1.976422000 0.300067000  6 1.643467000 0.562113000 0.143013000  6 0.479323000 -0.186109000 -0.235870000  6 0.616552000 -1.590391000 -0.401311000  6 1.844509000 -2.177771000 -0.178327000  6 2.933457000 -1.346316000 0.209584000  7 2.847515000 -0.043391000 0.362676000  1 -1.746989000 2.411709000 -0.485759000  1 0.282356000 3.692806000 0.189605000  1 2.455946000 2.515469000 0.588318000  1 3.913110000 -1.789697000 0.393838000  1 1.993675000 -3.248115000 -0.298419000  6 -3.564311000 -0.076026000 0.333753000  1 -4.170962000 0.199423000 -0.525037000  1 -3.198379000 0.742554000 0.952732000  6 -3.422127000 -1.419308000 0.736771000  1 -4.041845000 -2.178219000 0.262540000  1 -3.075385000 -1.625315000 1.748294000  1 -2.346070000 -1.198180000 -0.020796000  1 -0.242838000 -2.180413000 -0.712397000 |
| 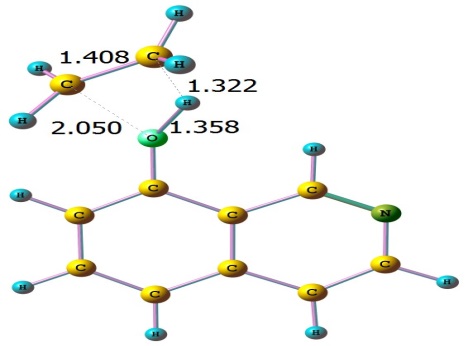  **TS12_enol_**  8 1.854069000 -0.147019000 -0.757672000  6 0.759302000 0.535484000 -0.441938000  6 0.788300000 1.921990000 -0.288902000  6 -0.380850000 2.634630000 0.082595000  6 -1.584241000 1.990568000 0.302242000  6 -1.656068000 0.576337000 0.144671000  6 -0.489145000 -0.160356000 -0.230938000  6 -0.613059000 -1.571567000 -0.395073000  7 -1.728114000 -2.245682000 -0.204614000  6 -2.832859000 -1.544291000 0.168426000  6 -2.850151000 -0.176700000 0.346311000  1 1.719519000 2.451939000 -0.477632000  1 -0.318871000 3.715989000 0.194195000  1 -3.768273000 0.330420000 0.637964000  6 3.570284000 -0.073746000 0.360981000  1 3.200178000 0.722932000 1.005323000  1 4.174516000 0.230638000 -0.489641000  6 3.416471000 -1.429387000 0.709207000  1 3.060689000 -1.674810000 1.708772000  1 4.029856000 -2.174542000 0.205741000  1 2.352266000 -1.184866000 -0.036616000  1 0.256394000 -2.148703000 -0.708803000  1 -2.475265000 2.544832000 0.589536000  1 -3.734653000 -2.135672000 0.319343000 | 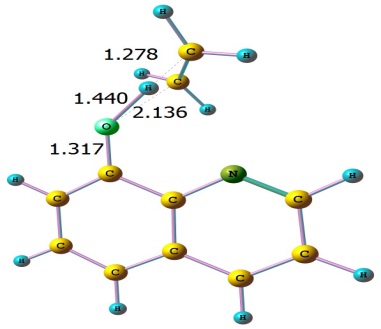  **TS13_enol_**  8 1.766019000 -1.101474000 -0.281421000  6 0.455856000 -1.143870000 -0.158081000  6 -0.230222000 -2.359313000 -0.086302000  6 -1.641490000 -2.411623000 0.035889000  6 -2.398382000 -1.256116000 0.089788000  6 -1.748359000 0.011197000 0.029670000  6 -0.320551000 0.083027000 -0.083643000  7 0.347662000 1.268870000 -0.122475000  6 -0.346996000 2.387012000 -0.070283000  6 -1.765561000 2.434229000 0.028370000  6 -2.459095000 1.244153000 0.081634000  1 0.355673000 -3.274295000 -0.138700000  1 -2.131657000 -3.382653000 0.082891000  1 -3.482624000 -1.295118000 0.178890000  1 -3.545652000 1.228814000 0.162887000  1 -2.275837000 3.393918000 0.063466000  1 0.223477000 3.316294000 -0.105557000  6 3.159955000 0.057155000 0.847945000  1 3.613359000 -0.855470000 1.225227000  1 2.378715000 0.517705000 1.447545000  6 3.602941000 0.671470000 -0.337555000  1 4.525942000 0.322031000 -0.796942000  1 3.301142000 1.699734000 -0.527675000  1 2.627101000 -0.045182000 -0.747931000 |

Table S2. IRC diagrams of for different transition states structures at BMK/6-31+G(d,p).

| **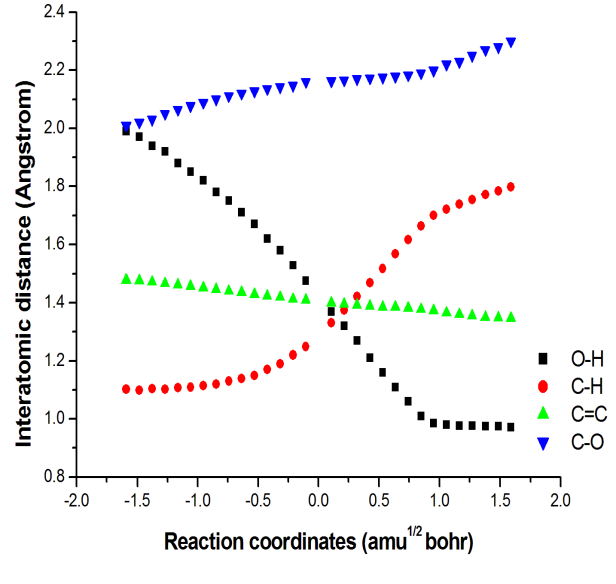** | **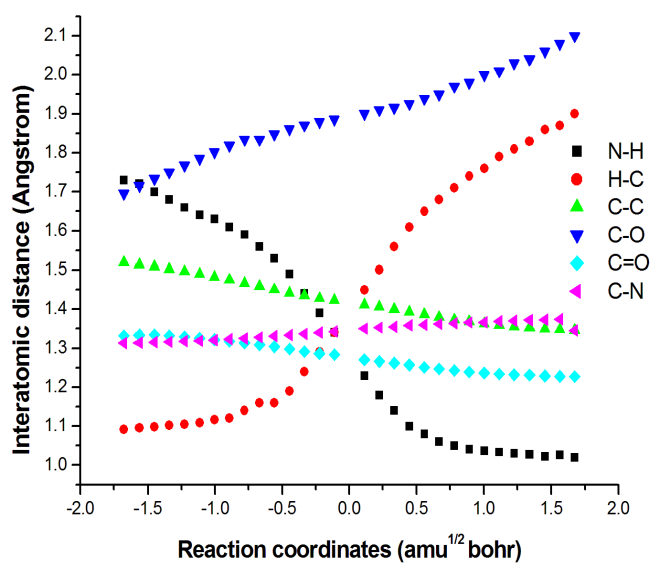** |
| --- | --- |
| **TS1** | **TS2** |
| **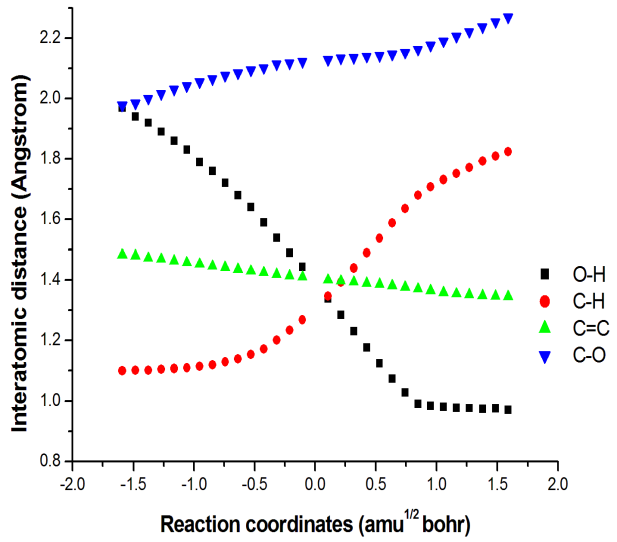** | **** |
| **TS3** | **TS4** |
| **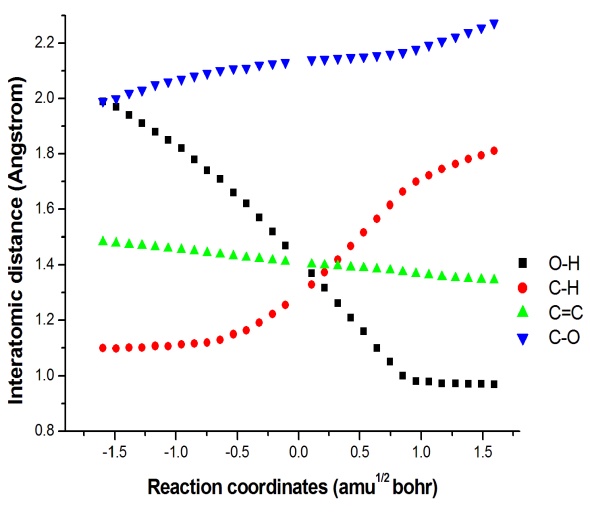** | **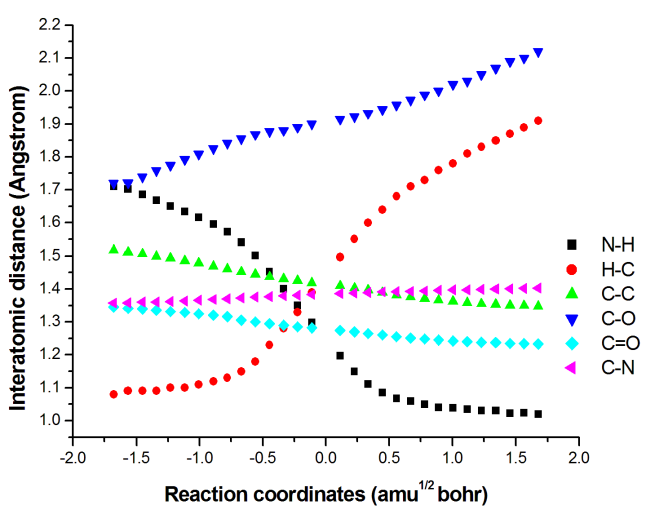** |
| **TS5** | **TS6** |
| **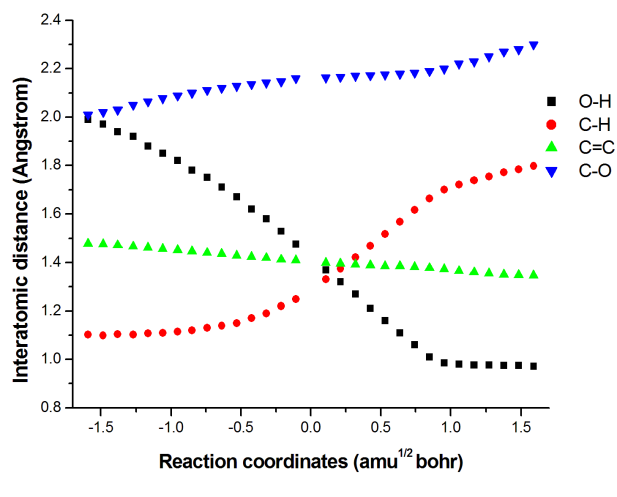** | **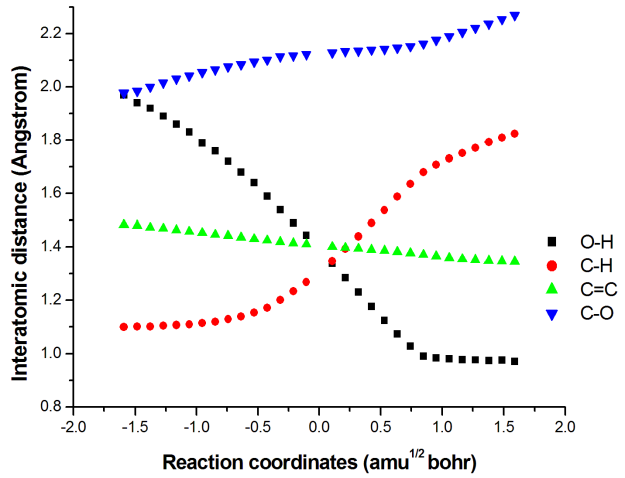** |
| **TS7** | **TS8** |
| **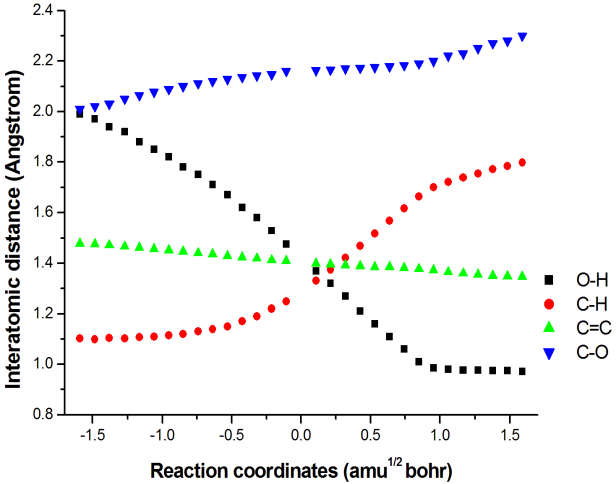** |  |
| **TS9** | **TS10** |
|  |  |
| **TS11** | **TS12** |
|  |  |
| **TS13** |  |

**Table S3:** Unimolecular rate constants *k*_1_ (s^-1^) of R1 for the reported **1-EisoQ** reaction channels obtained by means of TST, TST/Eck and RRKM theories using CBS-QB3 energies (*T* = 400–1200 K, *P* = 1 atm),

R1,k_1_, **1-EisoQ** → 1-HOisoQ + C_2_H_4_

| *T* K | Eck | TST | TST/Eck | RRKM |
| --- | --- | --- | --- | --- |
| 400 | 5.13 | 4.63E-23 | 2.38E-22 | 2.40E-22 |
| 500 | 2.49 | 9.49E-16 | 2.36E-15 | 2.38E-15 |
| 600 | 1.82 | 7.48E-11 | 1.36E-10 | 1.38E-10 |
| 700 | 1.53 | 2.45E-07 | 3.75E-07 | 3.79E-07 |
| 800 | 1.38 | 1.08E-04 | 1.49E-04 | 1.45E-04 |
| 900 | 1.29 | 1.24E-02 | 1.60E-02 | 1.64E-02 |
| 1000 | 1.23 | 5.61E-01 | 6.90E-01 | 6.94E-01 |
| 1100 | 1.18 | 1.29E+01 | 1.52E+01 | 1.53E+01 |
| 1200 | 1.15 | 1.76E+02 | 2.02E+02 | 2.02E+02 |

**Table S4:** Unimolecular rate constants *k*_2_ (s^-1^) for R2**,1-EisoQ** → 1-isoQO + C_2_H_4_

| *T* K | Eck | TST | TST/Eck | RRKM |
| --- | --- | --- | --- | --- |
| 400 | 4.74 | 3.64E-13 | 1.73E-12 | 1.73E-12 |
| 500 | 2.43 | 5.91E-08 | 1.44E-07 | 1.46E-07 |
| 600 | 1.8 | 1.83E-04 | 3.30E-04 | 3.36E-04 |
| 700 | 1.52 | 5.92E-02 | 9.00E-02 | 9.02E-02 |
| 800 | 1.37 | 4.60E+00 | 6.30E+00 | 6.33E+00 |
| 900 | 1.28 | 1.38E+02 | 1.76E+02 | 1.79E+02 |
| 1000 | 1.22 | 2.10E+03 | 2.57E+03 | 2.59E+03 |
| 1100 | 1.18 | 1.97E+04 | 2.33E+04 | 2.33E+04 |
| 1200 | 1.15 | 1.28E+05 | 1.48E+05 | 1.46E+05 |

**Table S5:** Unimolecular rate constants *k*_3_ (s^-1^) for R3. **2-EQ** → 2-HOQ + C_2_H_4_

| *T* K | Eck | TST | TST/Eck | RRKM |
| --- | --- | --- | --- | --- |
| 400 | 5.92 | 3.98E-23 | 2.36E-22 | 2.38E-22 |
| 500 | 2.66 | 8.57E-16 | 2.28E-15 | 2.28E-15 |
| 600 | 1.9 | 6.95E-11 | 1.32E-10 | 1.32E-10 |
| 700 | 1.58 | 2.32E-07 | 3.66E-07 | 3.67E-07 |
| 800 | 1.41 | 1.03E-04 | 1.46E-04 | 1.46E-04 |
| 900 | 1.31 | 1.21E-02 | 1.58E-02 | 1.59E-02 |
| 1000 | 1.24 | 5.54E-01 | 6.87E-01 | 6.88E-01 |
| 1100 | 1.2 | 1.27E+01 | 1.52E+01 | 1.52E+01 |
| 1200 | 1.16 | 1.74E+02 | 2.02E+02 | 2.02E+02 |

# Table S6: Unimolecular rate constants *k*_4_ (s^-1^) for R4. *k*_4_, 2-EQ → 2-QO + C_2_H_4_

| *T* K | Eck | TST | TST/Eck | RRKM |
| --- | --- | --- | --- | --- |
| 400 | 4.62 | 1.25E-13 | 5.76E-13 | 5.78E-13 |
| 500 | 2.41 | 2.40E-08 | 5.79E-08 | 5.83E-08 |
| 600 | 1.79 | 8.38E-05 | 1.50E-04 | 1.54E-04 |
| 700 | 1.52 | 2.92E-02 | 4.44E-02 | 4.48E-02 |
| 800 | 1.37 | 2.41E+00 | 3.30E+00 | 3.34E+00 |
| 900 | 1.28 | 7.54E+01 | 9.65E+01 | 9.69E+01 |
| 1000 | 1.22 | 1.20E+03 | 1.46E+03 | 1.47E+03 |
| 1100 | 1.18 | 1.16E+04 | 1.36E+04 | 1.39E+04 |
| 1200 | 1.15 | 7.70E+04 | 8.85E+04 | 8.89E+04 |

**Table S7:** Unimolecular rate constants *k*_5_ (s^-1^) for R5. *k*_5_, 3**-EisoQ** → 3-HOisoQ + C_2_H_4_

| *T* K | Eck | TST | TST/Eck | RRKM |
| --- | --- | --- | --- | --- |
| 400 | 5.64 | 6.81E-23 | 3.84E-22 | 3.84E-22 |
| 500 | 2.6 | 1.28E-15 | 3.33E-15 | 3.34E-15 |
| 600 | 1.87 | 9.43E-11 | 1.76E-10 | 1.77E-10 |
| 700 | 1.56 | 2.94E-07 | 4.59E-07 | 4.59E-07 |
| 800 | 1.4 | 1.25E-04 | 1.75E-04 | 1.77E-04 |
| 900 | 1.3 | 1.41E-02 | 1.83E-02 | 1.88E-02 |
| 1000 | 1.24 | 6.19E-01 | 7.68E-01 | 7.69E-01 |
| 1100 | 1.19 | 1.39E+01 | 1.66E+01 | 1.67E+01 |
| 1200 | 1.16 | 1.86E+02 | 2.16E+02 | 2.18E+02 |

**Table S8:** Unimolecular rate constants *k*_6_ (s^-1^) for R6. **3-EisoQ** → 3-isoQO + C_2_H_4_

| *T* K | Eck | TST | TST/Eck | RRKM |
| --- | --- | --- | --- | --- |
| 400 | 4.31 | 1.74E-15 | 7.50E-15 | 7.52E-15 |
| 500 | 2.32 | 8.11E-10 | 1.88E-09 | 1.89E-09 |
| 600 | 1.75 | 5.09E-06 | 8.91E-06 | 8.93E-06 |
| 700 | 1.5 | 2.70E-03 | 4.05E-03 | 4.07E-03 |
| 800 | 1.36 | 3.04E-01 | 4.14E-01 | 4.15E-01 |
| 900 | 1.27 | 1.23E+01 | 1.56E+01 | 1.56E+01 |
| 1000 | 1.21 | 2.38E+02 | 2.88E+02 | 2.89E+02 |
| 1100 | 1.17 | 2.71E+03 | 3.17E+03 | 3.18E+03 |
| 1200 | 1.14 | 2.06E+04 | 2.35E+04 | 2.36E+04 |

**Table S9:** Unimolecular rate constants *k*_7_ (s^-1^) for R7. **3-EQ** → 3-HOQ + C_2_H_4_

| *T* K | Eck | TST | TST/Eck | RRKM |
| --- | --- | --- | --- | --- |
| 400 | 7.56 | 8.36E-22 | 6.32E-21 | 6.34E-21 |
| 500 | 2.98 | 8.46E-15 | 2.52E-14 | 2.55E-14 |
| 600 | 2.03 | 4.16E-10 | 8.45E-10 | 8.48E-10 |
| 700 | 1.65 | 9.70E-07 | 1.60E-06 | 1.62E-06 |
| 800 | 1.46 | 3.30E-04 | 4.82E-04 | 4.82E-04 |
| 900 | 1.34 | 3.14E-02 | 4.21E-02 | 4.22E-02 |
| 1000 | 1.27 | 1.21E+00 | 1.54E+00 | 1.53E+00 |
| 1100 | 1.22 | 2.43E+01 | 2.96E+01 | 2.96E+01 |
| 1200 | 1.18 | 2.97E+02 | 3.50E+02 | 3.51E+02 |

**Table S10:** Unimolecular rate constants *k*_8_ (s^-1^) for R8, **4-EisoQ** → 4-isoHOQ + C_2_H_4_

| *T* K | Eck | TST | TST/Eck | RRKM |
| --- | --- | --- | --- | --- |
| 400 | 8.11 | 3.95E-21 | 3.20E-20 | 3.22E-20 |
| 500 | 3.07 | 2.81E-14 | 8.63E-14 | 8.63E-14 |
| 600 | 2.07 | 1.09E-09 | 2.26E-09 | 2.26E-09 |
| 700 | 1.68 | 2.14E-06 | 3.60E-06 | 3.62E-06 |
| 800 | 1.47 | 6.50E-04 | 9.55E-04 | 9.57E-04 |
| 900 | 1.35 | 5.60E-02 | 7.56E-02 | 7.56E-02 |
| 1000 | 1.28 | 1.99E+00 | 2.55E+00 | 2.56E+00 |
| 1100 | 1.22 | 3.76E+01 | 4.59E+01 | 4.62E+01 |
| 1200 | 1.18 | 4.36E+02 | 5.15E+02 | 5.18E+02 |

**Table S11:** Unimolecular rate constants *k*_9_ (s^-1^) for R9, **4-EQ** → 4-HOQ + C_2_H_4_

| *T* K | Eck | TST | TST/Eck | RRKM |
| --- | --- | --- | --- | --- |
| 400 | 6.27 | 1.93E-19 | 1.21E-18 | 1.22E-18 |
| 500 | 2.75 | 7.82E-13 | 2.15E-12 | 2.15E-12 |
| 600 | 1.93 | 2.10E-08 | 4.05E-08 | 4.05E-08 |
| 700 | 1.6 | 3.14E-08 | 5.03E-08 | 5.03E-08 |
| 800 | 1.42 | 7.82E-03 | 1.11E-02 | 1.12E-02 |
| 900 | 1.32 | 5.73E-01 | 7.56E-01 | 7.58E-01 |
| 1000 | 1.25 | 1.81E+01 | 2.26E+01 | 2.29E+01 |
| 1100 | 1.2 | 3.08E+02 | 3.69E+02 | 3.69E+02 |
| 1200 | 1.17 | 3.26E+03 | 3.81E+03 | 3.83E+03 |

**Table S12:** Unimolecular rate constants *k*_10_ (s^-1^) for R10. **5-EisoQ** → 5-isoHOQ + C_2_H_4_

| *T* K | Eck | TST | TST/Eck | RRKM |
| --- | --- | --- | --- | --- |
| 400 | 9.5 | 6.72E-22 | 6.38E-21 | 6.38E-21 |
| 500 | 3.28 | 9.12E-15 | 2.99E-14 | 2.99E-14 |
| 600 | 2.15 | 5.44E-10 | 1.17E-09 | 1.17E-09 |
| 700 | 1.72 | 1.47E-06 | 2.53E-06 | 2.53E-06 |
| 800 | 1.5 | 5.60E-04 | 8.40E-04 | 8.40E-04 |
| 900 | 1.37 | 5.84E-02 | 8.00E-02 | 8.00E-02 |
| 1000 | 1.29 | 2.42E+00 | 3.12E+00 | 3.12E+00 |
| 1100 | 1.23 | 5.15E+01 | 6.34E+01 | 6.34E+01 |
| 1200 | 1.19 | 6.63E+02 | 7.89E+02 | 7.89E+02 |

**Table S13:** Unimolecular rate constants *k*_11_ (s^-1^) for R11. *k_11_*, 5-EQ → 5-HOQ + C2H4

| *T* K | Eck | TST | TST/Eck | RRKM |
| --- | --- | --- | --- | --- |
| 400 | 9.57 | 2.78E-21 | 2.66E-20 | 2.67E-20 |
| 500 | 3.3 | 2.92E-14 | 9.62E-14 | 9.64E-14 |
| 600 | 2.16 | 1.47E-09 | 3.18E-09 | 3.18E-09 |
| 700 | 1.72 | 3.51E-06 | 6.03E-06 | 6.03E-06 |
| 800 | 1.5 | 1.22E-03 | 1.83E-03 | 1.84E-03 |
| 900 | 1.38 | 1.17E-01 | 1.62E-01 | 1.63E-01 |
| 1000 | 1.29 | 4.61E+00 | 5.95E+00 | 5.97E+00 |
| 1100 | 1.24 | 9.27E+01 | 1.15E+02 | 1.19E+02 |
| 1200 | 1.19 | 1.15E+03 | 1.37E+03 | 1.39E+03 |

**Table S14:** Unimolecular rate constants *k*_12_ (s^-1^) for R12, **8-EisoQ** → 8-isoHOQ + C_2_H_4_

| *T* K | Eck | TST | TST/Eck | RRKM |
| --- | --- | --- | --- | --- |
| 400 | 8.4 | 6.98E-22 | 5.86E-21 | 5.88E-21 |
| 500 | 3.12 | 9.13E-15 | 2.85E-14 | 2.86E-14 |
| 600 | 2.09 | 5.36E-10 | 1.12E-09 | 1.15E-09 |
| 700 | 1.68 | 1.42E-06 | 2.39E-06 | 2.39E-06 |
| 800 | 1.48 | 5.35E-04 | 7.92E-04 | 7.94E-04 |
| 900 | 1.36 | 5.49E-02 | 7.46E-02 | 7.46E-02 |
| 1000 | 1.28 | 2.26E+00 | 2.89E+00 | 2.89E+00 |
| 1100 | 1.22 | 4.78E+01 | 5.83E+01 | 5.84E+01 |
| 1200 | 1.19 | 6.06E+02 | 7.21E+02 | 7.25E+02 |

**Table S15:** Unimolecular rate constants *k*_13_ (s^-1^) for R13.

| *T* K | Eck | TST | TST/Eck | RRKM |
| --- | --- | --- | --- | --- |
| 400 | 4.33 | 5.80E-21 | 2.51E-20 | 2.51E-20 |
| 500 | 2.3 | 3.92E-14 | 9.01E-14 | 9.01E-14 |
| 600 | 1.73 | 1.48E-09 | 2.56E-09 | 2.56E-09 |
| 700 | 1.48 | 2.84E-06 | 4.21E-06 | 4.21E-06 |
| 800 | 1.35 | 8.37E-04 | 1.13E-03 | 1.13E-03 |
| 900 | 1.26 | 7.17E-02 | 9.04E-02 | 9.04E-02 |
| 1000 | 1.21 | 2.52E+00 | 3.05E+00 | 3.05E+00 |
| 1100 | 1.17 | 4.71E+01 | 5.51E+01 | 5.51E+01 |
| 1200 | 1.14 | 5.42E+02 | 6.18E+02 | 6.18E+02 |

**Table S16:** simple bond fissions rate constants *k*_14_*-k*_28_ (s^-1^) for R14-R28.

| *T* K | R14 | R15 | R16 | R17 | R18 | R19 | R20 | *R21* | *R22* | *R23* |
| --- | --- | --- | --- | --- | --- | --- | --- | --- | --- | --- |
| 400 | 2.19E-38 | 4.06E-29 | 3.88E-35 | 1.52E-38 | 2.52E-100 | 1.44E-40 | 5.40E-29 | 6.57E-37 | 4.55E-38 | 6.62E-59 |
| 500 | 1.95E-26 | 4.00E-19 | 2.33E-24 | 3.96E-27 | 4.46E-76 | 3.39E-28 | 4.79E-19 | 8.27E-26 | 1.02E-26 | 5.79E-43 |
| 600 | 1.76E-18 | 1.85E-12 | 3.70E-17 | 1.68E-19 | 6.22E-60 | 5.84E-20 | 2.05E-12 | 2.15E-18 | 3.98E-19 | 2.34E-32 |
| 700 | 8.21E-13 | 1.05E-07 | 5.23E-12 | 4.82E-14 | 2.02E-48 | 4.34E-14 | 1.11E-07 | 4.31E-13 | 1.07E-13 | 8.47E-25 |
| 800 | 1.42E-08 | 3.79E-04 | 3.89E-08 | 6.04E-10 | 8.41E-40 | 1.07E-09 | 3.85E-04 | 4.12E-09 | 1.27E-09 | 3.80E-19 |
| 900 | 2.74E-05 | 2.17E-01 | 3.98E-05 | 9.31E-07 | 4.15E-33 | 2.71E-06 | 2.14E-01 | 5.16E-06 | 1.89E-06 | 9.23E-15 |
| 1000 | 1.14E-02 | 3.44E+01 | 1.02E-02 | 3.31E-04 | 9.07E-28 | 1.40E-03 | 3.30E+01 | 1.55E-03 | 6.55E-04 | 2.89E-11 |
| 1100 | 1.55E+00 | 2.12E+03 | 9.51E-01 | 4.06E-02 | 2.09E-23 | 2.28E-01 | 2.00E+03 | 1.64E-01 | 7.78E-02 | 2.05E-08 |
| 1200 | 9.15E+01 | 6.49E+04 | 4.15E+01 | 2.21E+00 | 8.84E-20 | 1.57E+01 | 6.01E+04 | 8.02E+00 | 4.18E+00 | 4.79E-06 |

| *T K* | *R24* | *R25* | *R26* | *R27* | *R28* |
| --- | --- | --- | --- | --- | --- |
| *400* | 4.68E-37 | 3.96E-28 | 2.26E-34 | 1.90E-37 | 2.72E-58 |
| *500* | 2.18E-25 | 2.62E-18 | 9.59E-24 | 3.94E-26 | 1.77E-42 |
| *600* | 1.27E-17 | 9.27E-12 | 1.21E-16 | 1.41E-18 | 5.89E-32 |
| *700* | 4.36E-12 | 4.37E-07 | 1.46E-11 | 3.63E-13 | 1.85E-24 |
| *800* | 5.99E-08 | 1.37E-03 | 9.51E-08 | 4.18E-09 | 7.50E-19 |
| *900* | 9.67E-05 | 7.07E-01 | 8.86E-05 | 6.06E-06 | 1.67E-14 |
| *1000* | 3.47E-02 | 1.02E+02 | 2.10E-02 | 2.05E-03 | 4.90E-11 |
| *1100* | 4.19E+00 | 5.89E+03 | 1.84E+00 | 2.40E-01 | 3.30E-08 |
| *1200* | 2.24E+02 | 1.70E+05 | 7.62E+01 | 1.27E+01 | 7.35E-06 |

Table S17. Branching Ratios analysis for 1-EiSoQ, 2EQ and 3-EiSoQ using CBS-QB3 energies at 400-1200 K.

1. **1-EiSoQ**

| T | R1 | R2 | R14 | R15 | R16 | R17 | R18 |
| --- | --- | --- | --- | --- | --- | --- | --- |
| 400 | 0.00 | 100.00 | 0.00 | 0.00 | 0.00 | 0.00 | 0.00 |
| 500 | 0.00 | 100.00 | 0.00 | 0.00 | 0.00 | 0.00 | 0.00 |
| 600 | 0.00 | 100.00 | 0.00 | 0.00 | 0.00 | 0.00 | 0.00 |
| 700 | 0.00 | 100.00 | 0.00 | 0.00 | 0.00 | 0.00 | 0.00 |
| 800 | 0.00 | 99.99 | 0.00 | 0.01 | 0.00 | 0.00 | 0.00 |
| 900 | 0.01 | 99.87 | 0.00 | 0.12 | 0.00 | 0.00 | 0.00 |
| 1000 | 0.03 | 98.65 | 0.00 | 1.32 | 0.00 | 0.00 | 0.00 |
| 1100 | 0.06 | 91.58 | 0.01 | 8.35 | 0.00 | 0.00 | 0.00 |
| 1200 | 0.10 | 69.32 | 0.04 | 30.52 | 0.02 | 0.00 | 0.00 |

1. **2EQ**

| T | R3 | R4 | R19 | R20 | R21 | R22 | R23 |
| --- | --- | --- | --- | --- | --- | --- | --- |
| 400 | 0.00 | 100.00 | 0.00 | 0.00 | 0.00 | 0.00 | 0.00 |
| 500 | 0.00 | 100.00 | 0.00 | 0.00 | 0.00 | 0.00 | 0.00 |
| 600 | 0.00 | 100.00 | 0.00 | 0.00 | 0.00 | 0.00 | 0.00 |
| 700 | 0.00 | 100.00 | 0.00 | 0.00 | 0.00 | 0.00 | 0.00 |
| 800 | 0.00 | 99.98 | 0.00 | 0.01 | 0.00 | 0.00 | 0.00 |
| 900 | 0.02 | 99.76 | 0.00 | 0.22 | 0.00 | 0.00 | 0.00 |
| 1000 | 0.05 | 97.74 | 0.00 | 2.21 | 0.00 | 0.00 | 0.00 |
| 1100 | 0.10 | 87.15 | 0.00 | 12.75 | 0.00 | 0.00 | 0.00 |
| 1200 | 0.14 | 59.48 | 0.01 | 40.37 | 0.01 | 0.00 | 0.00 |

**(c)** **3-EiSoQ**

| T | R5 | R6 | R24 | R25 | R26 | R27 | R28 |
| --- | --- | --- | --- | --- | --- | --- | --- |
| 400 | 0.00 | 100.00 | 0.00 | 0.00 | 0.00 | 0.00 | 0.00 |
| 500 | 0.00 | 100.00 | 0.00 | 0.00 | 0.00 | 0.00 | 0.00 |
| 600 | 0.00 | 100.00 | 0.00 | 0.00 | 0.00 | 0.00 | 0.00 |
| 700 | 0.01 | 99.98 | 0.00 | 0.01 | 0.00 | 0.00 | 0.00 |
| 800 | 0.04 | 99.63 | 0.00 | 0.33 | 0.00 | 0.00 | 0.00 |
| 900 | 0.11 | 95.55 | 0.00 | 4.34 | 0.00 | 0.00 | 0.00 |
| 1000 | 0.20 | 73.67 | 0.01 | 26.12 | 0.01 | 0.00 | 0.00 |
| 1100 | 0.18 | 34.90 | 0.05 | 64.85 | 0.02 | 0.00 | 0.00 |
| 1200 | 0.11 | 12.14 | 0.12 | 87.59 | 0.04 | 0.01 | 0.00 |

**Table S18:** Comparsion of the calculated and experimental rate constants of R2, R4, and R6 using CBS-QB3 energies (experimental results from ReF 14).

| T | 1000/T | k2-EXP[14] | Log (K2-Exp) | K2 | Log (K2) | k4-EXP[14] | Log (K4-Exp) | K4 | Log (K4) | k6-EXP[14] | Log (K6-Exp) | K6 | Log (K6) |
| --- | --- | --- | --- | --- | --- | --- | --- | --- | --- | --- | --- | --- | --- |
| 649.9 | 1.539 | 0.0016 | -2.80 | 0.0067 | -2.17 | 0.00077 | -3.12 | 3.17E-03 | -2.50 | 0.00005 | -4.32 | 2.37E-04 | -3.63 |
| 664.5 | 1.505 | 0.0037 | -2.43 | 0.0148 | -1.83 | 0.00184 | -2.74 | 7.12E-03 | -2.15 | 0.00012 | -3.91 | 5.65E-04 | -3.25 |
| 678.7 | 1.473 | 0.0079 | -2.10 | 0.0312 | -1.51 | 0.00394 | -2.40 | 1.51E-02 | -1.82 | 0.00027 | -3.57 | 1.27E-03 | -2.90 |
| 698.5 | 1.432 | 0.0213 | -1.67 | 0.0838 | -1.08 | 0.01030 | -1.99 | 4.12E-02 | -1.39 | 0.00075 | -3.13 | 3.74E-03 | -2.43 |
| 712.9 | 1.403 | 0.0399 | -1.40 | 0.1660 | -0.78 | 0.02030 | -1.69 | 8.26E-02 | -1.08 | 0.00153 | -2.82 | 7.89E-03 | -2.10 |

**Table S19:** Unimolecular rate constants *k*_1_-_13_ (s^-1^) for R1-R13 at *T* = 800 K and *P* = 10^-6^ to 10 atm using RRKM.

| *P* (atm) | 1.00E-06 | 1.00E-05 | 1.00E-04 | 1.00E-03 | 1.00E-02 | 1.00E-01 | 1.00E+00 | 1.00E+01 |
| --- | --- | --- | --- | --- | --- | --- | --- | --- |
| *k*_1_ (s^-1^) | 2.28E+00 | 4.84E+00 | 7.50E+00 | 8.99E+00 | 9.42E+00 | 9.49E+00 | 9.50E+00 | 9.50E+00 |
| *k*_2_ (s^-1^) | 3.31E+02 | 1.76E+03 | 6.84E+03 | 1.77E+04 | 2.93E+04 | 3.48E+04 | 3.59E+04 | 3.61E+04 |
| *k*_3_(s^-1^) | 5.36E+00 | 1.42E+01 | 2.72E+01 | 3.81E+01 | 4.29E+01 | 4.41E+01 | 4.42E+01 | 4.42E+01 |
| *k*_4_ (s^-1^) | 3.60E+02 | 2.05E+03 | 8.79E+03 | 2.59E+04 | 4.94E+04 | 6.42E+04 | 6.82E+04 | 6.88E+04 |
| *k*_5_ (s^-1^) | 2.57E+00 | 5.69E+00 | 9.17E+00 | 1.13E+01 | 1.20E+01 | 1.21E+01 | 1.21E+01 | 1.21E+01 |
| *k*_6_ (s^-1^) | 1.93E+02 | 9.07E+02 | 3.08E+03 | 7.01E+03 | 1.07E+04 | 1.22E+04 | 1.25E+04 | 1.25E+04 |
| *k*_7_ (s^-1^) | 1.03E+01 | 2.82E+01 | 5.49E+01 | 7.74E+01 | 8.70E+01 | 8.92E+01 | 8.94E+01 | 8.95E+01 |
| *k*_8_ (s^-1^) | 1.52E+01 | 4.33E+01 | 8.69E+01 | 1.24E+02 | 1.41E+02 | 1.44E+02 | 1.45E+02 | 1.45E+02 |
| *k*_9_ (s^-1^) | 2.10E+01 | 6.18E+01 | 1.28E+02 | 1.86E+02 | 2.13E+02 | 2.18E+02 | 2.19E+02 | 2.19E+02 |
| *k*_10_ (s^-1^) | 1.72E+01 | 5.02E+01 | 1.03E+02 | 1.52E+02 | 1.74E+02 | 1.79E+02 | 1.79E+02 | 1.79E+02 |
| *k*_11_ (s^-1^) | 3.94E+01 | 1.45E+02 | 3.91E+02 | 7.41E+02 | 1.01E+03 | 1.11E+03 | 1.13E+03 | 1.13E+03 |
| *k*_12_ (s^-1^) | 1.68E+01 | 4.88E+01 | 1.00E+02 | 1.47E+02 | 1.68E+02 | 1.72E+02 | 1.73E+02 | 1.73E+02 |
| *k*_13_ (s^-1^) | 2.78E+01 | 8.98E+01 | 2.07E+02 | 3.34E+02 | 4.05E+02 | 4.24E+02 | 4.27E+02 | 4.28E+02 |
